# Networks of Neuronal Genes Affected by Common and Rare Variants in Autism Spectrum Disorders

**DOI:** 10.1371/journal.pgen.1002556

**Published:** 2012-03-08

**Authors:** Eyal Ben-David, Sagiv Shifman

**Affiliations:** Department of Genetics, The Institute of Life Sciences, The Hebrew University of Jerusalem, Jerusalem, Israel; Georgia Institute of Technology, United States of America

## Abstract

Autism spectrum disorders (ASD) are neurodevelopmental disorders with phenotypic and genetic heterogeneity. Recent studies have reported rare and *de novo* mutations in ASD, but the allelic architecture of ASD remains unclear. To assess the role of common and rare variations in ASD, we constructed a gene co-expression network based on a widespread survey of gene expression in the human brain. We identified modules associated with specific cell types and processes. By integrating known rare mutations and the results of an ASD genome-wide association study (GWAS), we identified two neuronal modules that are perturbed by both rare and common variations. These modules contain highly connected genes that are involved in synaptic and neuronal plasticity and that are expressed in areas associated with learning and memory and sensory perception. The enrichment of common risk variants was replicated in two additional samples which include both simplex and multiplex families. An analysis of the combined contribution of common variants in the neuronal modules revealed a polygenic component to the risk of ASD. The results of this study point toward contribution of minor and major perturbations in the two sub-networks of neuronal genes to ASD risk.

## Author Summary

Autism spectrum disorders (ASD) are neurodevelopmental syndromes with a strong genetic basis, but are influenced by many different genes. Recent studies have identified multiple genetic risk factors, including rare mutations and genetic variations common in the population. To identify possible connections between different genetic risk factors, we constructed a network based on the expression pattern of genes across different brain areas. We identified groups of genes that are expressed in a similar pattern across the brain, suggesting that they are involved in the same processes or types of cells. We found that the genetic risk factors were enriched in specific groups of connected genes. Of these, the strongest enrichment was discovered in a group of neuronal genes that are involved in processes of learning and memory, and are highly expressed during infancy. Further study of this group of genes has the potential to reveal a more detailed picture of the neuronal mechanisms leading to ASD and to provide knowledge required for developing diagnostic tools and effective therapies.

## Introduction

Autism is the most severe end of a group of neurodevelopmental disorders referred to as autism spectrum disorders (ASDs). ASD is a heterogeneous genetic syndrome characterized by social deficits, language impairments and repetitive behaviors. Although it is known that ASD has a genetic basis [1]–[3], its genetic architecture is unclear. Previous studies have identified both common and rare variants, including *de novo* mutations, as risk factors for ASD [4], [5]. However, how much of the genetic risk can be attributed to rare versus common alleles is unknown. Since ASD is relatively common with a complex pattern of inheritance it was previously suggested to be caused by multiple common variants [4], [5], where each of the common variants only makes a small contribution to the risk of disease. The principal methods for discovering common variations related to ASD include association studies of candidate genes, and more recently genome-wide association studies (GWAS) [6], [7]. Despite major efforts to identify common variants associated with ASD, the success so far has been limited [6], [7]. At the same time, an increasing number of studies have shown that rare and *de novo* mutations contribute to ASD [8]–[12]. These rare variants include mutations causing single-gene disorders, cytogenetically visible chromosomal abnormalities, and more recently the identification of rare and *de-novo* copy number variations (CNVs) [8]–[10], [12]. The genes already known to be disrupted by rare variants still account for only a small proportion of the cases, because many of them have only been found in one or very few individuals [13]. Other findings that further complicate the interpretation and utilization of rare variants is the fact that many of the same variants have been found in patients with distinct illnesses (such as schizophrenia, epilepsy, and intellectual disability), as well as in healthy family members or controls [14].

This genetic heterogeneity constitutes a considerable obstacle to establishing a thorough understanding of the etiology of ASD. One promising avenue of exploration is to find key molecular pathways and apply system-wide approaches to determine the function of the genes disrupted in ASD. Delineating these pathways will not only lead to insights into the molecular basis of ASD, but may ultimately lead to potential treatments. Most attempts so far have concentrated on determining the functional connection between genes affected by CNVs. These studies showed that many of the genes are related to synapse development, cellular proliferation, neuronal migration and projection [15], [16]. Another way to identify the connection between autism susceptibility genes is based on studying protein interactions for genes mutated in syndromes associated with autism [17]. This study suggested that shared molecular pathways are implicated in different ASD associated syndromes [17]. A different approach to identify key molecular pathways is based on gene expression, and relies on the assumption that co-expressed genes are functionally related [18]. A weighted gene co-expression network analysis (WGCNA) of specific human brain regions (cerebral cortex, cerebellum and caudate nucleus) demonstrated that the transcriptome of the human brain is organized into modules of co-expressed genes that reflect different neural cell types [19]. Recently, this type of analysis was also applied to compare the expression profiles of three brain regions from autistic and control individuals. This network analysis led to the identification of specific co-expression modules that are differentially expressed in ASD and controls [20]. These included a neuronal module that was enriched for genes with low GWAS *P*-values, suggesting that the differential expression of this module between cases and controls reflects a causal relationship [20].

In the current study, we constructed a gene network using a WGCNA approach based on a widespread survey of gene expression undertaken by the Allen Human Brain Atlas project (http://www.brain-map.org). This survey of gene expression provides unprecedented coverage across different brain regions. We found modules which are associated with specific neural cell types, and modules with highly significant enrichment for specific cellular processes. We used the gene network to address several fundamental questions regarding the genetic architecture of autism. First, can we identify gene networks that are perturbed by rare variations that in turn lead to ASD? Second, can we identify gene networks that are perturbed by common variations? Third, do rare and common variations converge on the same molecular pathways or do they represent diverse biological etiologies? Lastly, can we integrate the gene network with GWAS results to predict potential genes associated with ASD? To answer these questions we integrated the co-expression network with the results of autism GWAS and with known rare mutations. We identified specific modules that are enriched for both rare and common variations that are potentially associated with ASD risk. We replicated the enrichment in two additional samples. The modules showing the highest enrichment for rare and common variants in ASD included highly connected genes that are involved in synaptic and neuronal plasticity, and are expressed in areas associated with learning and memory and sensory perception. Additionally, we found that a genetic risk score based on these modules significantly predicts ASD risk. Taken together, these results suggest a common role for rare and common variations in autism, and illustrate how rare and *de novo* mutations, in conjunction with common variations, can act together to perturb gene networks involved in neuronal processes, and specifically neuronal plasticity. Furthermore, the modules found in this study may serve as starting points for designing potential therapeutic interventions for ASD.

## Results

### Network analysis of brain transcriptome identifies modules representing specific cell types and molecular functions

**Figure 1:**
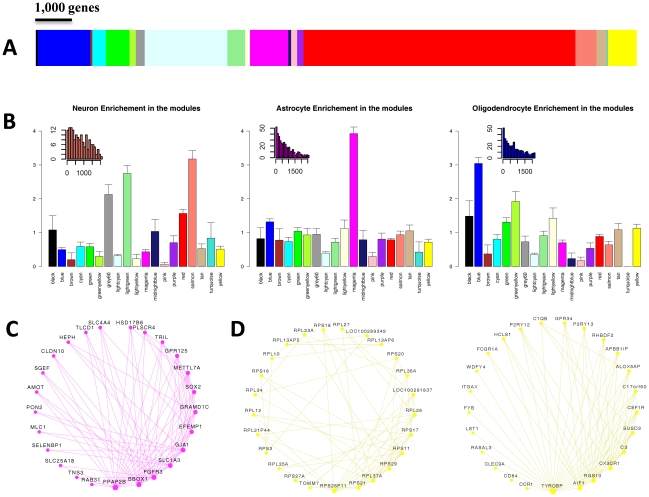
Weighted gene co-expression network analysis (WGCNA) of human brain transcriptomes. (A) Color map showing the relative sizes of the different modules in the WGCNA analysis. Colors correspond to arbitrary names given to each module. (B) Enrichment for genes up-regulated in Neurons (left), Astrocytes (center) and Oligodendrocytes (right). For each module, the relative risk (RR) for harboring cell-type specific genes is plotted. Insets show the distribution of the rank of enriched genes located in modules with the highest cell-type specific RR. The distribution is shown for the Salmon, Magenta and Blue module for genes enriched in neurons, astrocytes, and oligodendrocytes (respectively). (C) Top connected genes in the Magenta module. Nodes are ordered and sized according to their degree. (D) Top connected genes in the Yellow module, revealing two distinct groups of genes. One group (left circle) includes genes involved in protein translation, and the other (right circle) includes Microglia specific genes.

In order to construct a robust network of the human brain transcriptome we used the Allen Brain Atlas RNA microarray data, which to the best of our knowledge, is one of the most comprehensive expression profiling of different regions of the human brain. The Allen Brain Atlas RNA microarray data includes 1340 measurements from two individuals, representing the entirety of the adult human brain. We generated a network based on a combined dataset, as the two individuals exhibited high correlations in trends of expression and connectivity (Figure S1). The network included 19 modules of varying sizes, from 38 to 7385 genes (Figure 1A, Table S1). The different modules are color-coded for presentation purposes and referred to hereafter based on these colors (Figure 1A). To study the modules specificity to brain areas, we plotted the modules eigengenes across different anatomical regions, and observed that none of the modules were specific to one anatomical region (Figure S2, Table S2). We hypothesized that the modules may correspond to cell types or subcellular compartments, which are distributed in different densities across different brain areas. We thus tested the modules for enrichment of specific neural cell populations based on gene expression levels in neurons, astrocytes and oligodendrocytes, as found in a survey performed on mouse brain cells [21]. One module, Magenta, stood out as showing a very high enrichment for genes up-regulated in astrocytes (relative risk [RR] = 3.93, *P*<0.0001) (Figure 1B). Three other modules showed enrichment for genes up-regulated in neurons (Figure 1B). Of these, Salmon showed the highest enrichment signal (RR = 3.18 P<0.0001), in addition to two other modules, Lightgreen and Grey60, that also showed substantial enrichment for neuronal genes (RR = 2.75 and RR = 2.16, respectively, with *P*<0.0001 in both). Enrichment for genes up-regulated in oligodendrocytes was found in the Blue module (RR = 3.04, P<0.0001), and the Greenyellow module (RR = 1.92, P<0.0001) (Figure 1B). To test whether the modules were specifically enriched for the most representative genes of each cell type, we used a score of the relative expression in a particular cell type relative to other cells (Figure S3). Notably, the modules with the strongest enrichment for genes expressed in neurons, astrocytes and oligodendrocytes showed specific enrichment for the most up-regulated genes in the corresponding cell types (Figure S3). We tested the degree of overlap between these cell type-specific modules and ones that were discovered in a previous study that constructed a gene co-expression network that was largely based on differences between individuals rather than between brain areas [19]. The general comparison of the two networks is described in Table S3. A significant overlap in gene content between the studies was observed for an oligodendrocyte module (Blue, RR = 5.29, *P*<2×10^−5^) and the astrocyte module (Magenta, RR = 5.62, *P*<2×10^−5^). Similarly, the Salmon module significantly overlapped with a previously identified cortical module (RR = 9.73, *P*<2×10^−5^) and the Grey60 module showed a high overlap with a parvalbumin-expressing cortical interneuron module (RR = 83.44 *P*<2×10^−5^). The module Lightgreen had no significant overlap with any of the previously identified modules.

To further characterize the different modules we used gene ontology (GO) analysis (Table S4). The Salmon module was enriched for genes active in the synapse (*P* = 2.2×10^−6^) and involved in synaptic transmission (*P* = 4.8×10^−3^), as well as for genes in the calmodulin-binding pathway (*P* = 9.9×10^−4^). The Lightgreen module was also enriched for genes active in the synapse (*P* = 1.6×10^−5^). The GO analysis also showed a different module, Black, to be highly enriched for genes in the nucleosome core (*P* = 1×10^−31^). The representative of the gene expression profile of the Black module (the module eigengene) had the highest values in the corpus callosum and cingulum bundle, suggesting that this module may represent enrichment for cell bodies of glia cells (Table S5). In the Red module the genes having a positive relationship with the module eigengene were enriched for mitochondrion (*P* = 2.9×10^−40^), and the genes having a negative relationship were enriched for DNA binding (*P* = 6.6×10^−23^) and regulation of transcription (*P* = 2.2×10^−21^). Another module, Pink, was highly enriched for genes containing a Kruppel-Associated Box domain (*P* = 2.2×10^−46^). This group of zinc finger transcription factors has been recognized as transcriptional repressors [22]. The Tan module was highly enriched for genes involved in the G-protein-coupled receptor pathway (*P* = 2.6×10^−50^), as well for genes involved in olfactory receptor activity (*P* = 2.2×10^−42^), hormonal activity (*P* = 1.1×10^−28^) and HOX genes (1.6×10^−11^).

Another way to infer the function of the modules is based on the known function of highly connected genes with central positions within the modules (“hub” genes). We explored the strongest connections in each module using Cytoscape software [23] (Figure S4). In the Magenta module, which was found to be highly enriched for genes up-regulated in astrocytes, the most connected gene was *FGFR3*, which was reported to mark astrocytes and their neuroepithelial precursors in the CNS [24] (Figure 1C). The Yellow module, which was highly significantly enriched for genes involved in protein translation (*P* = 7.4×10^−98^), presented as two separate sub-networks of genes (Figure 1D). One group of highly connected genes is involved in protein translation, and the other group contains genes related to the function of microglia (Figure 1D). The central components of the microglia sub-network include *TYROBP*, *AIF1*, *RGS10*, *CX3CR1*, as well as other genes which are known to be involved in microglia function and regulation [25]–[27]. These results suggest that the module is representative of microglia which also show high protein translation associated with their high proliferation rate. Consistent with this observation, the module eigengene of the Yellow module was most highly expressed in the corpus callosum, where immature microglial progenitor cells accumulate [28], [29].

**Figure 2:**
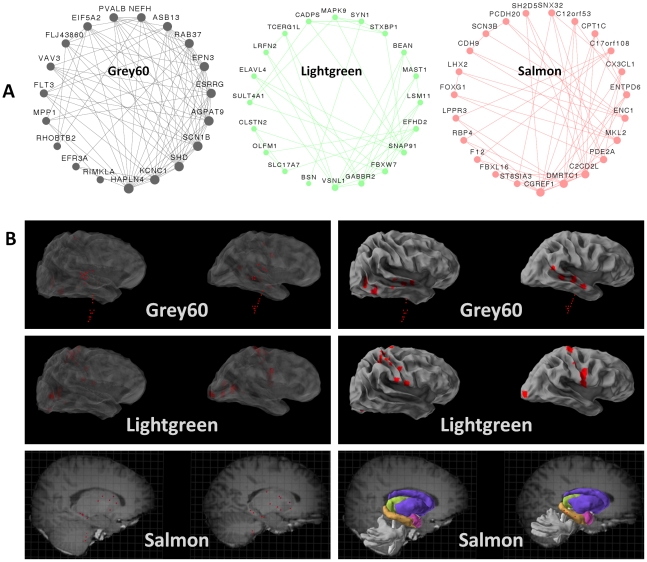
Modules correspond to specific neuronal sub-groups acting in specific regions. (A) For the three neuronal modules, the twenty genes with the most connections out of the top 150 connections in the module are illustrated. (B) For the three neuronal modules, the ten areas showing the highest expression of the module eigengene were visualized using Brain Explorer (http://www.brain-map.org). A superficial view of the brain is shown on the right, and an in-depth view is shown on the left. As some regions were available for only one of the two individuals, the regions are shown in both brains.

Given that our analysis highlighted three groups of neuronal genes, the next step was to determine whether they represent three different types of neurons. To that end, we visualized the top connections in the three modules (Figure 2A), and highlighted the brain areas showing the highest values for the first principal component of each module (the module eigengene) (Figure 2B). The top connections in one module (Grey60) included the genes *KCNC1*, *SCN1B*, *PVALB* and *HAPLN4* (Figure 2A). These genes have been shown to be highly expressed in a group of fast-spiking, parvalbumin-expressing cortical interneurons [30], [31]. The module was most expressed in the superior temporal gyrus, an area that receives auditory signals from the cochlea [32], the dentate nucleus, which is a structure linking the cerebellum to the rest of the brain [33], and the dorsal lateral geniculate nucleus, which is the primary relay center for visual information [34]. The eigengene of the Lightgreen module was most expressed in brain regions involved in sensory processes, including the inferior occipital gyrus and the lingual gyrus of the occipital lobe (Figure 2B), which are involved in processing visual information [35], [36], and the post central gyrus, which contains the primary somatosensory cortex [37]. The module Lightgreen harbors highly connected genes involved in clathrin-dependent endocytosis in the synapse. These include *SNAP91* (also known as *AP180*), *VSNL1* (also known as *VILIP-1*), *SYN1* and and *STXBP1* [38]–[41]. The Salmon module included several highly connected genes (*FOXG1*, *LHX2*, *MKL2*, *CDH9* and genes of the protocadherin family), which are all known to be involved in neurogenesis and neuronal plasticity in the developing brain [42]–[47]. *FOXG1*, *MKL2* and *PCDH20* have also been shown to be involved in structural and functional plasticity of neurons in the adult brain [48]–[50]. Similarly, the eigengene of the Salmon module was most expressed in brain regions that are involved in learning and memory, including the hippocampus (dentate gyrus and CA1 field) and the dorsal striatum (tail of the caudate nucleus and putamen) (Figure 2B).

### Rare and common genetic risk variants are significantly enriched in specific neuronal modules

**Figure 3:**
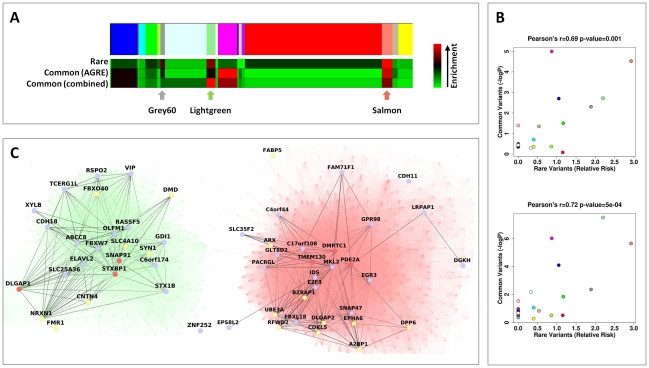
Rare and common variations in ASD perturb shared neuronal modules. (A) Color map showing the different modules in the WGCNA analysis. Below it, heat maps depicting, for each module, the relative risk (RR) for harboring genes with rare mutations (top), the enrichment for low GWAS p-values in the AGRE cohort (center) and the combined enrichment for low GWAS p-values across the three GWAS (bottom). The intensity of the red hue corresponds to higher enrichment of common or rare variants. (B) For each module, the enrichment for low GWAS *P*-values (−log_10_ *P*) in the AGRE cohort (top) or combined across the three cohorts (bottom) is plotted against the relative risk for rare mutations. The color of the points corresponds to the names of the modules. (C) The connections in the two neuronal modules enriched for common and rare variations, Lightgreen (left) and Salmon (right), are visualized. Three highly connected genes in each module (hub genes) are shown in red; genes with a gene-wide p-value<0.05 in light blue, and genes with known rare mutations in autism in yellow.

We sought to test whether autism genes affected by rare or spontaneous mutations are associated with specific modules. A list of 246 autism susceptibility genes was compiled using the SFARI gene database (https://sfari.org/sfari-gene), and was restricted to the 121 genes with reported rare mutations in autism. Of these, 91% (109 genes) were represented in our network. Genes on the list exhibited a significantly skewed distribution between the modules (*P* = 0.025, Fisher's test). Specifically, three modules showing up-regulation in neurons also showed the highest enrichment for autism risk genes. The most enriched module was the Salmon (RR = 2.92), followed by Lightgreen (RR = 2.19) and Grey60 (RR = 1.89) (Figure 3A). To test whether CNVs also tended to be distributed in a non-random way among modules, we assembled a list of *de-novo* CNV events from a recent study [10], and calculated enrichment to specific modules. As larger genes can be expected to harbor more CNVs by chance, and since neuronal specific genes are larger than average [51], we corrected for gene size in our analysis (see Materials and Methods). However, none of the modules showed significant enrichment for CNV events after correcting for gene size.

Subsequently, we tested the distribution across modules of genes affected by common variants, as reflected by low *P*-values in a GWAS for autism, previously performed [6] on multiplex families (with more than one member of the family with ASD) from the Autism Genetic Resource Exchange (AGRE) (Figure 3A). Notably, two of the three neuronal modules (Salmon and Lightgreen), which also showed the highest enrichment for genes affected by rare variants, were also found to be significantly enriched for genes affected by common variants (Salmon, *P* = 0.000030; Lightgreen, *P* = 0.0019; Bonferroni corrected *P*<0.05). The enrichment in the third neuronal module (Grey60) was not significant after correcting for multiple tests (nominal *P* = 0.005, Bonferroni corrected *P* = 0.095). In addition to the neuronal modules, significant enrichment was found in the astrocyte-associated Magenta module (*P*<0.00001) and the oligodendrocyte associated Blue module (nominal *P* = 0.0008, Bonferroni corrected *P* = 0.015).

We next examined the correlation between the degree of enrichment of rare and common variants for the different modules. Strikingly, the overall propensity to harbor genes with common variants enriched in autism, and the overall propensity to harbor genes with rare mutations linked to autism, were significantly correlated (Pearson correlation r = 0.69, *P* = 0.0010) (Figure 3A, 3B). Specifically, two of the three modules representing neuronal genes (Lightgreen and Salmon) were significantly enriched for genes affected by both rare and common variations, with the highest overall evidence for association in the Salmon module. As can be seen in Figure 3C, the genes affected by common and rare variants within the Lightgreen and Salmon modules are highly interconnected.

Differences in transcriptome organization between autistic and normal brain have been recently reported, including a neuronal module associated with ASD [20]. To study how the enrichment of rare and common variants corresponded to this study, we tested the overlap between the neuronal modules obtained in our study and the neuronal module that was previously shown to be differentially expressed between cases and controls [20]. Interestingly, the highest overlap was observed with the Grey60 module (RR = 6.18), followed by Lightgreen (RR = 4.59), but there was only relatively minor overlap with the Salmon module (RR = 1.84).

To test the robustness of the enrichment of GWAS low p-values in specific modules we first applied the same analysis on GWAS data for type-2-diabetes [52]. The analysis with type-2-diabetes did not reveal any association with the modules. Next, we attempted to replicate the results in two additional GWAS of ASD. The first is a previously reported [53] GWAS from the Autism Genome Project (AGP), which includes both multiplex and simplex families (around 40% of families had two or more ASD children). The second is based on genotyping data of simplex families (with a single child with ASD) from the Simons Simplex Collection (SSC). Inherited and *de novo* CNVs were previously reported for this sample [10], but no genome-wide association for common variants was reported. We performed a genome-wide association using the transmission disequilibrium test (TDT). To reduce the genetic heterogeneity, in both datasets we focused on families with European ancestry (Figure S5A). Quantile-quantile (Q-Q) plots showed that there was minimal inflation of the test statistics (genomic control inflation factor for AGP *λ*_GC_ = 1.0268, for SSC *λ*_GC_ = 1.0013) (Figure S5B). None of the SNPs in the SSC cohort were genome-wide significant (P<5×10^−8^). The 10 most significant SNPs in the SSC GWAS are shown in Table S6. We also examined the 29 SNPs that were proposed as possible ASD risk variants by previous genome-wide studies [6], [7], [53] (Table S7). Of these, 22 were either available in our data or had a proxy SNP with an R^2^>0.8. None of the 22 SNPs were associated in the SSC cohort (all *P*>0.05).

Despite the limited results when testing single SNPs by association, the enrichment of low p-values in specific modules was replicated across different GWAS. The enrichment in the neuronal modules, Salmon and Lightgreen, was replicated both in the AGP (Salmon, *P* = 0.012; Lightgreen, *P* = 0.000057) and in the SSC GWAS (Salmon, *P* = 0.033; Lightgreen, *P* = 0.0026). The combined p-value for low p-values enrichment, across the three studies, was 2.2×10^−6^ for the Salmon module, and 7.3×10^−8^ for the Lightgreen module. In addition, a replication of the enrichment of low p-values was obtained for the Blue and Magenta modules using the results of the AGP GWAS (Blue, *P* = 0.014; Magenta, *P* = 0.0011), but not with the SSC (Blue, *P* = 0.062; Magenta, *P* = 0.43). Based on the three genome-wide studies the most enriched module for common risk variants is the Lightgreen module, while the Salmon is the module most enriched for rare variants (Figure 3A). However, the correlation between the enrichment of rare variants and common variants (based on the three GWAS together) remained significant (r = 0.72, *P* = 5×10^−4^) (Figure 3B).

To identify candidate genes central to the enrichment for common variants, we calculated a gene-wide *P*-value for association with ASD for all genes that contributed to the enrichment score in the three samples (showing overrepresentation of low GWAS *P*-values in the modules). Eighty five genes passed a cutoff of 0.05 for gene-wide significance in one of the studies (Table S8). Out of these 85 genes, SNPs in four genes (DMD, ATP2B2, MACROD2 and MKL2) were previously found to be associated with ASD [53]–[56].

**Figure 4:**
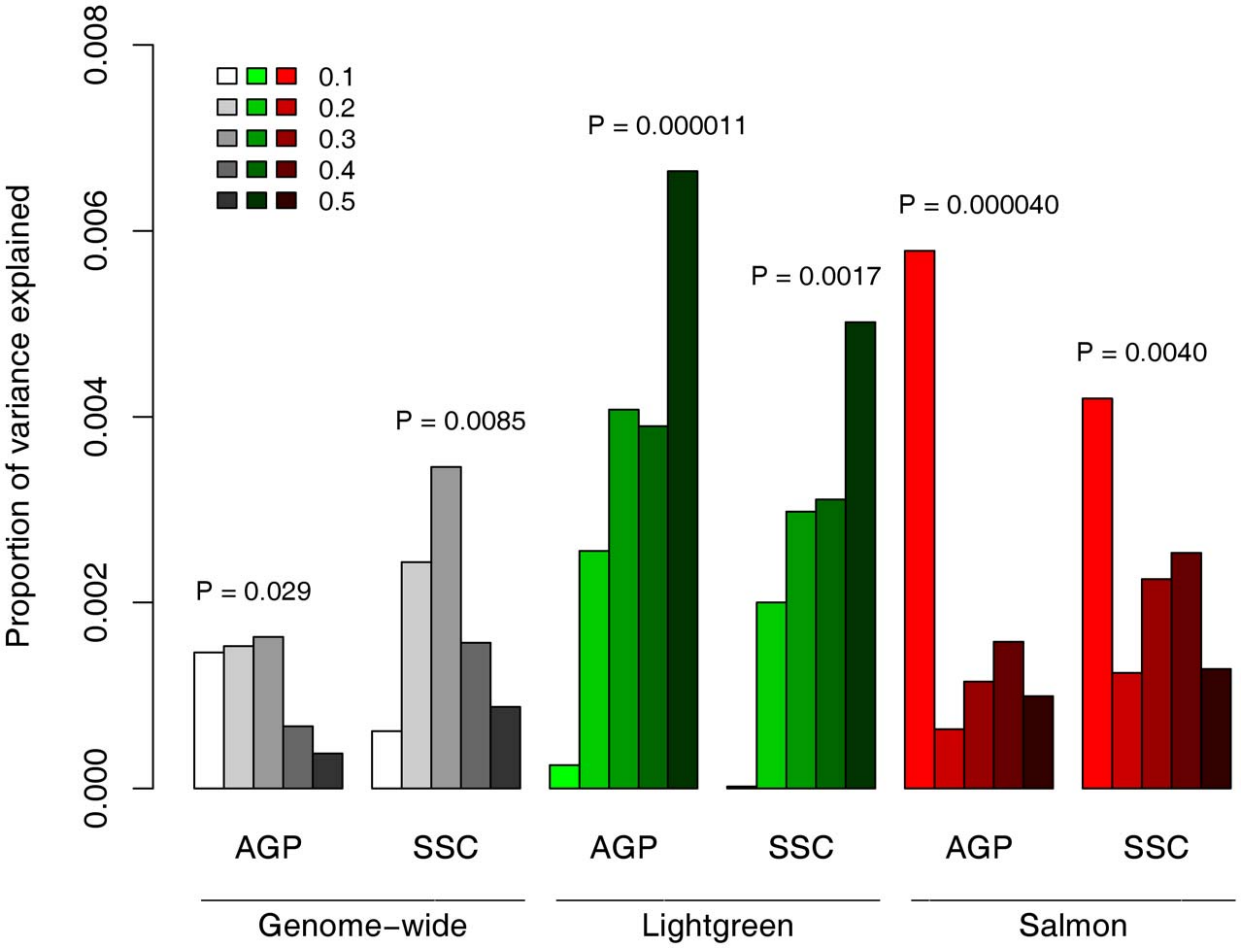
Contribution to ASD risk of common variation in the neuronal sub-networks. Genetic risk scores were calculated for each individual in the AGP and SSC cohorts, based on the genotypes of 79,079 tag SNPs and the association signal in these SNPs in the AGRE cohort. These scores were used to predict ASD status in a logistic regression model. To test the Lightgreen and Salmon modules, the analysis was limited to tag SNPs residing in genes in these modules. In each module and cohort, different series denote different thresholds of P-values, from P_T_<0.1 to P_T_<0.5.

The replicated enrichment suggests that multiple common variants, particularly in sub-networks of neuronal genes, contribute collectively to ASD risk. This raised the possibility that common variants within the two neuronal modules may specifically predict ASD risk. If the observed enrichment is specific to ASD, one would expect that a score that incorporates the effect of multiple SNPs would be a significant predictor of ASD risk. To test this, we performed a genetic risk score analysis based on 79,079 tag SNPs (as previously reported [57]). The AGRE dataset served as the discovery sample. We selected SNPs at different thresholds of association p-values (P_T_), and based on whether they belong to the neuronal modules, Salmon or Lightgreen. Based on the GWAS results in the AGRE, we calculated a genetic score for each individual in the AGP or SSC samples and tested whether the score can predict ASD status. While a marginally significant correlation was observed between the score and diseases status with genome-wide data (P_T_<0.3, AGP, *P* = 0.029; SSC, *P* = 0.0085), the score based on the neuronal modules was highly significant (Figure 4). The score based on SNPs in the Lightgreen module had increased association with ASD risk with more liberal thresholds in both AGP and SSC samples, with 0.66–0.5% (respectively) of the variance explained at the threshold of P_T_<0.5 (AGP, *P* = 1.1×10^−5^; SSC, *P* = 0.0017). Strikingly, a very different pattern was observed for the Salmon module: the strongest association, in both AGP and SSC, was with the strictest threshold of P_T_<0.1 (AGP, *P* = 4.0×10^−5^; SSC, *P* = 0.0040).

**Figure 5:**
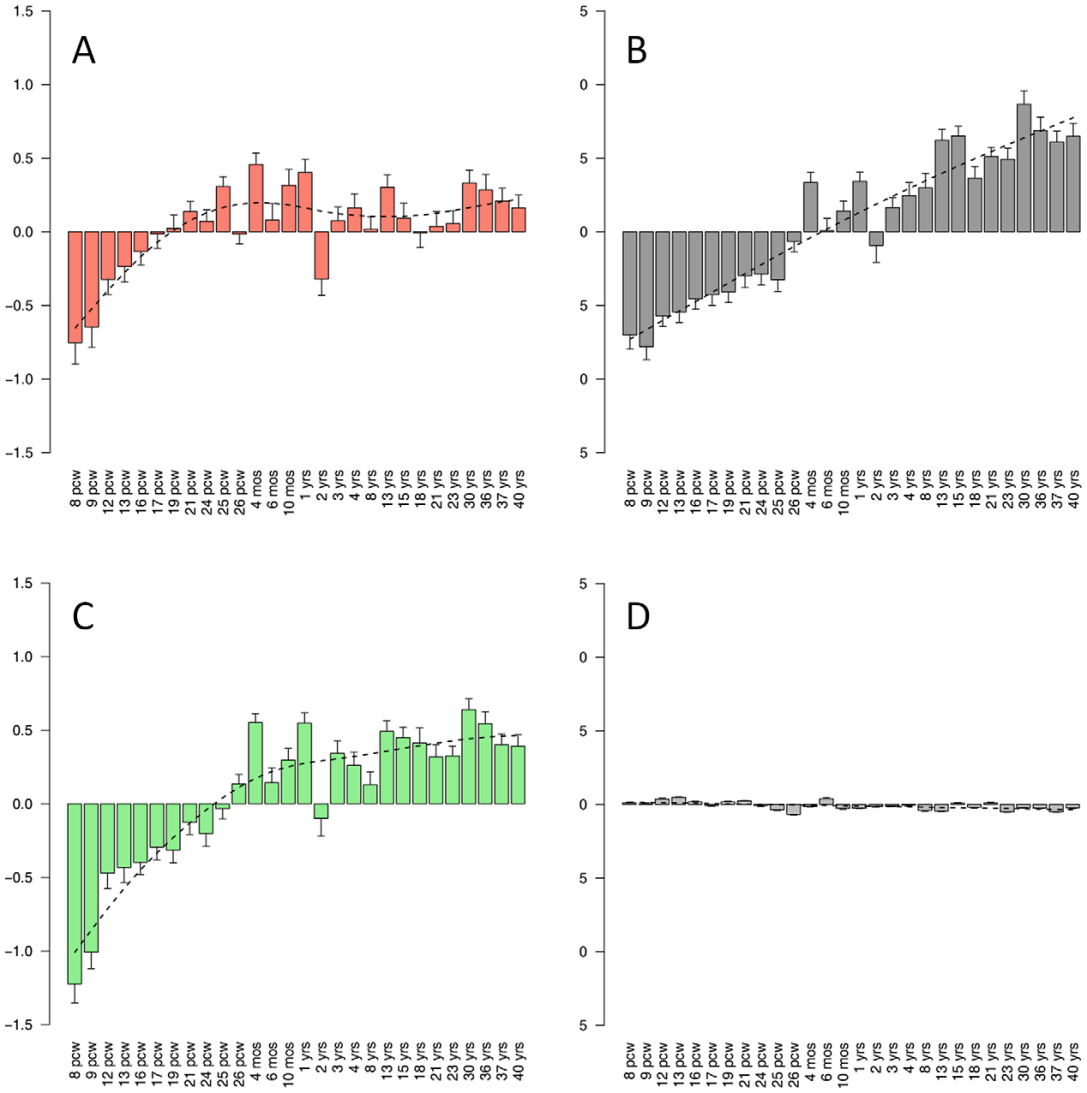
Expression of neuronal modules during developmental stages. The temporal expression pattern is plotted for the modules Salmon (A), Grey60 (B) and Lightgreen (C), along with the pattern of the entire gene set (D). For each module, the average normalized expression across different brain regions for the 50 most connected genes is shown in different ages, overlaid by a smoothed signal (dashed line). pcw, post-conceptional weeks; mos, months; yrs, years.

The Salmon module, which is one of the enriched modules for ASD risk variants, includes genes that are known to be expressed in both the developing and the adult brain. This raises the question of whether this module represents pathways that are mainly involved in neuronal plasticity in the adult brain, or whether it represents genes that operate mainly in the developing brain. To address this question we examined gene expression profiles of brain samples from different developmental stages, using data from the BrainSpan database. For each of the neuronal modules we calculated the average expression for the 50 most connected genes across different brain areas, and plotted this as a function of developmental stages (Figure 5). In all three neuronal modules there was a relatively low expression during fetal brain development that increased with fetal age. In the Salmon module the highly connected genes showed the highest expression during infancy (Figure 5A). In contrast, in the Grey60 module, which represents genes expressing in cortical interneurons, there was a continuous increase into adulthood (Figure 5B). The most connected genes in the Lightgreen module had, on average, a relatively stable expression from childhood to adulthood (Figure 5C). A flat temporal pattern was observed for the entire dataset of genes (Figure 5D).

## Discussion

We constructed a gene co-expression network based on comprehensive expression profiling of the human brain. The network was based on the variation in expression between different brain regions. Similar to the findings of a previous study [19], modules in the network corresponded to specific cell types. The vastness of the data allowed us to detail various cell-types, and even, in the case of neurons and oligodendrocytes, to identify modules corresponding to sub-populations of cells. Furthermore, functional annotation of the modules allowed us to characterize genes related to specific cellular processes and molecular functions in the brain, which in many cases (but not all) are also related to specific cell populations. These modules, and the hierarchy of the genes within them (especially the “hub” genes), can be used to predict the function of yet uncharacterized genes and learn about new biological phenomena. An intriguing example is the observed coupling between two sub-networks within the Yellow module. One sub-network corresponds to genes involved in protein translation and the other to microglia function and regulation. This module's eigengene can be used to estimate the relative distribution of microglia in the brain. Another example is the identification of three separate neuronal modules, suggesting that the neurons in the brain could be roughly divided into three main types based on their gene expression profiles. One module corresponds to fast-spiking Parvalbumin-expressing interneurons. These interneurons have been shown to be of importance in the generation of gamma-oscillations [58], which are required for speech perception and production [59], [60], consistent with the strong signal of the module eigengene in the temporal cortex. The observed increase in the expression of the genes in this module with age is consistent with previous reports in human and rats [61], [62]. The second module is involved in sensory perception. Accordingly, it is highly expressed in the visual and somatosensory cortices and enriched with synaptic genes. The third module includes genes implicated in neuronal plasticity, and is highly expressed in brain areas responsible for learning and memory. Similarly, we identified two modules that are enriched for genes up-regulated in oligodendrocytes, the Blue and Yellowgreen modules. The Yellowgreen module was also found to be enriched for genes involved in mitosis and the cell cycle. We suggest that the Blue module may represent mature oligodendrocytes, whereas the Yellowgreen module might represent immature dividing cells.

An important route in utilizing this network is as a framework to explore the functional aspects of genetic variations in brain related phenotypes. Because the network is based on measurements from control individuals alone, it can only shed light on diseases where specific aspects of brain functionality are involved. Our focus in this study was ASD, as this is a heterogeneous syndrome with a diverse genetic contribution. Although the genetic architecture of ASD is still under debate, we found enrichment of genes affected by both common and rare variants within specific neuronal modules. The enrichment of genes affected by common variants was replicated in two additional samples. Furthermore, we found a genetic risk score based on the two neuronal modules to be significantly associated with ASD status in the two target samples. The replication was evident despite the fact that the discovery sample consists mainly of multiplex families and the target samples of only simplex families or a mix of both. GWAS for ASD have had limited success so far; however, our study suggests a polygeneic component of ASD risk that is shared by multiplex and simplex families. This implies that a GWAS with larger samples should further contribute to the identification of ASD susceptibility genes. The effect of multiple common variants with very low effect size perturbs neuronal sub-networks, which are also affected by rare variants. With this in mind, it is tempting to speculate that both common and rare variants contribute to perturbations of the same neuronal pathways, which in turn lead to ASD.

Unlike genome-wide studies of SNPs and CNVs that aim to identify specific genes associated with ASD, the approach used here seeks to identify sub-networks that have a causal relationship with ASD. Nevertheless, by integrating the network and the GWAS data, we were able to elucidate genes within the modules that are more likely to be responsible for the observed enrichment, and are thus likelier candidates for association with ASD, needing further validations. One of the sub-networks that are enriched for rare and common variants (the Salmon module) represents genes that are expressed in neurons, and are related to neuronal plasticity and neurogenesis. Accordingly, the expression of genes in this module was highest in the dentate gyrus, the CA1 field of the hippocampus, and the dorsal striatum. By examining expression levels during different developmental stages, we found that the highest expression of the most connected genes in the Salmon module was during infancy. The other associated module (Lightgreen) is enriched with synaptic genes, specifically genes involved in clathrin-dependent endocytosis, with the highest expression in cortical areas involved in sensory processes. While this could reflect the involvement of these regions with ASD etiology, it is important to note that our findings could reflect the distribution of specific cell types in the adult brain, and not necessarily the brain areas affected in ASD.

The results of this study are in line with previous findings that connected rare mutations in autism with neuronal activity-dependent genes [63]. The hypothesis is that these genes are highly expressed during critical periods of infancy and early childhood as they are influenced by neural activity, which is dependent on inputs from the environment [64]. Perturbation by common and rare variants in these genes and pathways that are involved in learning and memory of social cues during postnatal stages may increase the risk of developing ASD. The potential involvement of postnatal neuronal plasticity in ASD gives hope that these pathways may be amenable to treatment long after symptom onset, as has been suggested by animal studies on various neurodevelopmental syndromes [65], [66].

In summary, we constructed a gene network based on comprehensive expression profiling of the human brain. This network can be used as a framework to study multiple questions including ones related to disease mechanisms, but also to normal functions of genes in the brain. It could also be integrated with other functional assays of the brain or other datasets. In our current study we focused on ASD as a case study. We integrated the gene co-expression network with genetic variations associated with ASD. The results support the notion that common and rare variants contribute to ASD by perturbation of common neuronal networks. Further integration of genetic and molecular data with the network has the potential to reveal a more detailed picture of the particular molecular features depicted in the network that contribute to ASD. Such knowledge is essential, first for providing insights into the molecular functionality related to the etiology of ASD, and also for the development of diagnostic tools and effective therapies.

## Materials and Methods

### Dataset summary

Microarray data were acquired from the Allen Brain Atlas (http://human.brain-map.org/well_data_files), and included a total of 1340 microarray profiles from donors H0351.2001 and H0351.2002, encompassing the different regions of the human brain. Donors were 24 and 39 years old, respectively, with no known psychopathologies. For donor H0351.2001 a total of 921 microarray profiles were available, and for H0351.2002 a total of 419 microarray profiles were available. A detailed description of the donor individuals, including available medical profile and post-mortem analyses performed is available at the following link:http://help.brain-map.org/download/attachments/2818165/CaseQual_and_DonorProfiles_WhitePaper.pdf. A detailed description of the regions measured by microarray in each donor is available in Table S9.

### WGCNA network

Statistical analysis was done using the R project for statistical computing (http://www.r-project.org). Network construction deployed the WGCNA R-package [67], and followed closely the tutorials available on the authors' website. First, the correlation between both individuals was tested by correlating first the mean rank of the expression values in each gene, and then by correlating the mean connectivity values in each gene [68]. For genes with at least 3 available probes, the connectivity for each of the probes was calculated, and the probe with the highest connectivity was chosen for the network analysis. For genes with 2 probes, the one with the highest mean was chosen. Probes not corresponding to refSeq genes were removed, leaving a total of 16,298 probes used in the network. The network was assembled following previously published parameters [69]. An adjacency matrix was calculated by raising the correlation matrix by a power of 6 (determined to be optimal for scale free topology in our dataset), and a TOM matrix was generated [20]. To determine the modules, hierarchical clustering was performed, and the tree was cut using the cutreeHybrid function in the WGCNA R package, with the minimum module size set to 30 genes, and parameter deepSplit set to 2 [67]. The resultant modules were merged using the mergeCloseModules function with cutHeight set to 0.3. The module eigengenes were derived by taking the 1^st^ principal component in a PCA analysis for the expression values in each module. To visualize the modules, the 150 strongest connections were drawn in the Cytoscape software [23]. For presentation purposes, the nodes were ordered based on their degree of connectivity, and their number was restricted to 50 nodes in each module.

### Enrichment for neural cell types

The enrichment analysis was based on a dataset of genes enriched in mouse neurons, oligodendrocytes and astrocytes [21]. First, the number cell-type enriched genes in each module (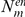) was calculated, as well as the total number of cell-type enriched genes (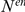) appearing in the entire network. Subsequently, a Relative Risk (RR) measure was calculated for each module and for each cell type, 
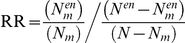
, with 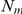 as the number of genes in each module, and 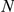 the total number of genes in the network. *P*-values were obtained by permutation testing, whereby a module of the same size was randomly selected and the RR calculated. Standard error was calculated for each module using bootstrap analysis. To determine whether the observed overall enrichment was specific to the more up-regulated genes in the cell types, the distribution of rank fold-change for the cell-type enriched genes in each module was plotted. The correlation between the median of the bins in the histogram and the number of genes in the bins was tested. A strong negative correlation indicates a substantial enrichment of the higher ranked cell type specific genes.

### Gene ontology (GO) enrichment analysis

Lists of the genes in each module were tested with the DAVID bioinformatics tool [70]. For background, the complete list of the genes in the network was used. For the module red, due to its size, the 3000 genes with the highest correlation with the module eigengene (see above) and the 3000 genes with the lowest correlation (most negative) with the module eigengene were used separately for enrichment testing.

### Rare mutation and copy number variation (CNV) analysis

A list of autism susceptibility genes was compiled using the SFARI gene database (https://sfari.org/sfari-gene), downloaded on the 23/6/2011. The list was restricted to genes with reported rare mutations in autism. Fisher's exact test was used to test the distribution of ASD genes within the modules with 10,000 permutations. The number of risk genes with rare variants (from the SFARI Gene) in each module (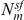), and the total number of risk genes in the network (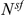), were used to calculate the RR, similar to the method described above: 
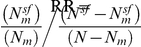
. For the CNV analysis, gene length was used instead of gene count, to correct for biases arising from differences in gene lengths between the modules. For each module, the total length in base pairs covered by CNV (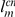)and the total length of the module (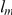), were used along with the total length covered by CNV (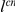) and the total length of the genes in the network (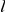), in the following formula: 
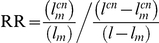
.

### Enrichment for low GWAS p-values

Testing for the enrichment of low GWAS *P*-values was performed using the discovery cohort of a previously published GWAS [6], which included 943 ASDs families. The analysis incorporated a previously published method [71], in a manner previously described [20]. Briefly, the minimum *P*-value for each gene was used in an enrichment score similar to the Kolmogorov-Smirnov statistic [71]. Gene boundaries included the 20 kb upstream and 10 kb downstream of each gene. To arrive at a *P*-value corrected for the size of the genes, the gene labels were permuted. Permutations were run until either reaching 20 instances of the higher enrichment score, or 100,000 permutations.

For each of the three neuronal modules found to be enriched for rare and common variations in ASD, a list of genes that contributed positively to the enrichment score of the module was obtained. As the enrichment for low GWAS p-values was tested using a running sum statistic over a sorted gene list, all genes above the point where the statistic reached the maximum were taken. Gene-wide *P*-values were determined by taking the SNP with the minimum *P*-value in each gene and correcting for the number of SNPs in the gene using a Bonferroni correction.

### Replication of enrichment for low GWAS p-values in additional samples

All GWAS analyses were performed using the PLINK software by Shaun Purcell [72]. SNP genotyping data was acquired from the Simons Simplex Collection (SSC) and the Autism Genome Project (AGP). The SSC cohort included 734 nuclear families with an autistic proband and an unaffected sibling, along with two parents, genotyped using the Illumina 1M platform. The AGP cohort included 1369 nuclear families with an autistic proband and two parents, genotyped using the Illumina 1M platform. To determine divergent ancestry, each sample separately was combined with data from The HapMap Phase III, following a previously published procedure [73]. Multidimensional scaling analysis to four dimensions was then performed in PLINK, followed by clustering to four groups using the R Package Mclust [74]. After removing individuals who did not cluster with the Hapmap CEU cohort, 588 families remained in the SSC cohort, and 1165 families remained in the AGP cohort. On these, TDT was performed, limiting the analysis to SNPs with a minor allele frequency of over 10%, in Hardy-Weinberg Equilibrium (P>0.001 in an exact test), with more than 90% genotyping rate, and with less than 10% rate of mendelian errors. This left 788010 SNPs in the SSC and 668221 in the AGP. Families with 5% mendelian errors were set to be removed, but none crossed that threshold. Q-Q plots were generated by plotting the observed −log_10_ *P* against the expected distribution, and visualized using a function available online. (http://gettinggeneticsdone.blogspot.com/2011/04/annotated-manhattan-plots-and-qq-plots.html).

### Estimation of the contribution of common variation to Autism

To estimate the contribution of common variation to autism, we followed a previously published paradigm [57]. First, a list of tag SNPs was compiled wherein no two SNPs had an r^2^>0.25 in a combined SSC and AGP sample. For these SNPs, the z-score of the reference allele for association in the AGRE cohort was used to calculate a score in Plink for each individual, which was defined as the sum across all SNPs of the number of reference allele multiplied by the z-score. The predictive value of the score was tested by fitting a logistic regression model with ASD status as the explained variable and individual score as the predictor, and calculating both a Wald's test p-value and a Nagelkerke's pseudo-r^2^. To test the Salmon and Lightgreen modules, the list of tag SNPs was further pruned for SNPs in genes in these modules, and the same analysis was performed.

### Analysis of gene expression during brain development

Gene expression microarray profiles of the brain from individuals of different ages were retrieved from the BrainSpan database (http://developinghumanbrain.org/). The data included 492 microarray measurements from a total of 35 individuals of 28 different ages, ranging from 8 weeks post-conception to 40 years of age (full sample information is available on the BrainSpan website). We first accounted for global differences between the different array samples and between the different genes. For each measurement (*a*) of a gene (*i*) in each array (*j*), the following compound z-score was calculated: 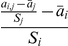. As several array measurements from different brain regions existed for each age, the mean normalized score was used in the final analysis. For each module tested, the mean score of the 50 genes with the most connections out of the top 150 connections was plotted. A smoothed signal was calculated using the cubic smoothing spline algorithm implemented in the R function *smooth.spline*, using default parameters.

## Supporting Information

Figure S1 High correlation in trends of (A) expression and (B) connectivity between the two individuals (9861, 10021). The rank of the mean expression (A) and connectivity (B) were calculated for each gene and each individual. (A) Ranked gene expression values in individual #9861 as a function of the values in individual #10021 (each point is a different gene). (B) Ranked connectivity values for each gene in individual #9861 as a function of the value in individual #10021.
(PDF)

Figure S2 Expression patterns of the modules eigengene across brain regions. For each module in the network, the level of the module eigengene is shown for 100 representative regions. The 100 regions were chosen using the following algorithm: first, the region showing the highest standard deviation was chosen, and then the 99 additional regions were chosen by taking recursively the region showing the lowest r^2^ with the previous ones.
(PDF)

Figure S3 Enrichment for specific cell types in the different modules. Histogram of the rank of enrichment score for genes found to be enriched in (A) neurons, (B) astrocytes, and (C) oligodendrocytes is plotted for each module. Higher density towards the lower end of the spectrum denotes enrichment for the higher ranked cell-type genes. Pearson correlations along with P-values are listed below each plot. (A) The Salmon and Lightgreen modules show highly significant enrichment for the higher ranking of neuronal specific genes. (B) The Magenta module shows highly significant enrichment for the higher ranking astrocyte specific genes. (C) The Blue module shows highly significant enrichment for the high ranking oligodendrocyte specific genes.
(PDF)

Figure S4 Top connections in the WGCNA modules. The top 150 connections in each module are visualized using the Cytoscape Software Package. Nodes are ordered and sized according to their degree.
(PDF)

Figure S5 Quality control measures for GWAS. MDS clustering (A) and Q-Q (B) plots are shown for the AGP (left) and SSC (right) cohorts. (A) An MDS plot was generated incorporating samples from the HapMap Project Phase III. On this, clustering was performed using the Mclust R package. Samples (shown in gray) which did not cluster with the HapMap Caucasian (CEU) cohort (shown in turquoise) were removed from analysis. Han Chinese and Japanese samples are in purple, and Yorubans are in yellow. (B) Q-Q plot was generated by plotting the observed −log_10_ *P* against the expected under a uniform P-value distribution.
(PDF)

Table S1 List of genes in the WGCNA network, their module affiliation and their degree of membership to each module.
(CSV)

Table S2 The gene expression profile of each module, represented by the module eigengene, is shown for different brain regions and samples.
(XLSX)

Table S3 Overlap of the gene co-expression network results with a previously published network.
(XLSX)

Table S4 Enrichment of gene ontology (GO) categories in the different modules.
(XLSX)

Table S5 For each module, a list of the top 10 brain regions with highest level of expression based on the module eigengene.
(XLSX)

Table S6 Top GWAS association signals in the SSC sample.
(XLSX)

Table S7 Replication attempts of published GWAS results in the SSC and AGP samples.
(XLSX)

Table S8 A list of candidate genes with gene-wide *P*-value<0.05, which contributed to the enrichment of low GWAS p-values in the Lightgreen and Salmon modules.
(XLSX)

Table S9 A list of brain regions with gene expression microarray used to construct the gene co-expression network.
(XLSX)
