# Supplementary material for: Networks of Neuronal Genes Affected by Common and Rare Variants in Autism Spectrum Disorders
Source: PLoS Genet. 2012 Mar 8;8(3):e1002556. doi: 10.1371/journal.pgen.1002556 (PMC3297570; doi:10.1371/journal.pgen.1002556)
Supplement: Figure S1 — High correlation in trends of (A) expression and (B) connectivity between the two individuals (9861, 10021). The rank of the mean expression (A) and connectivity (B) were calculated for each gene and each individual. (A) Ranked gene expression values in individual #9861 as a function of the values in individual #10021 (each point is a different gene). (B) Ranked connectivity values for each gene in individual #9861 as a function of the value in individual #10021. (PDF) [file pgen.1002556.s001.pdf]

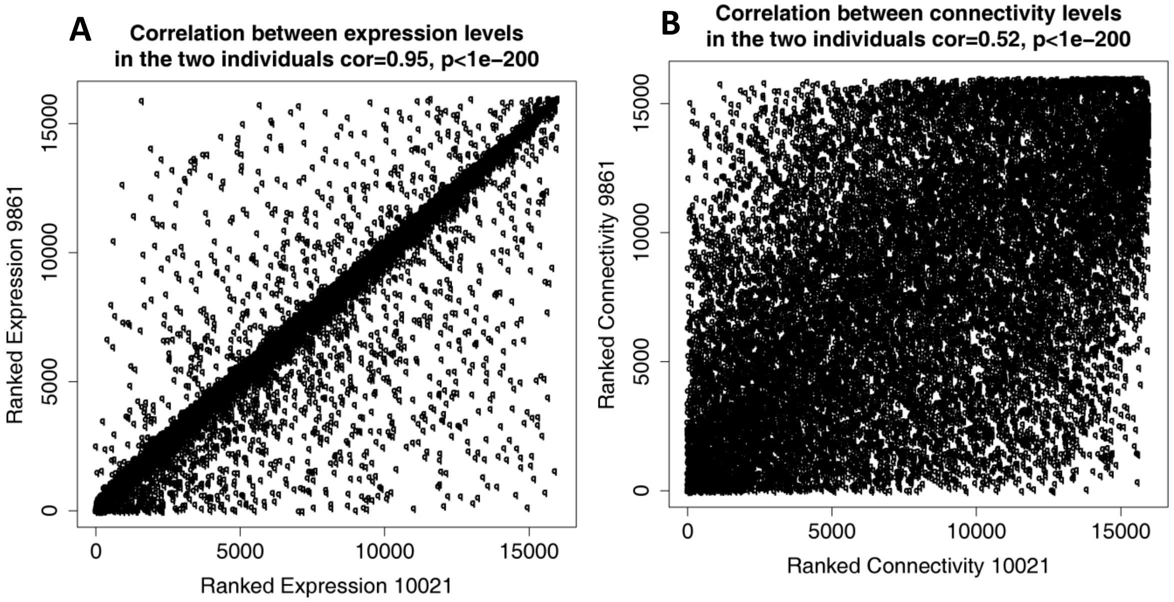
A
Correlation between expression levels
in the two individuals cor=0.95, p<1e−200
Ranked Expression 9861
Ranked Expression 10021
0
5000
10000
15000
B
Correlation between connectivity levels
in the two individuals cor=0.52, p<1e−200
Ranked Connectivity 9861
Ranked Connectivity 10021
0
5000
10000
15000
