# Supplementary material for: Networks of Neuronal Genes Affected by Common and Rare Variants in Autism Spectrum Disorders
Source: PLoS Genet. 2012 Mar 8;8(3):e1002556. doi: 10.1371/journal.pgen.1002556 (PMC3297570; doi:10.1371/journal.pgen.1002556)

(A) Enrichment for neuronal genes

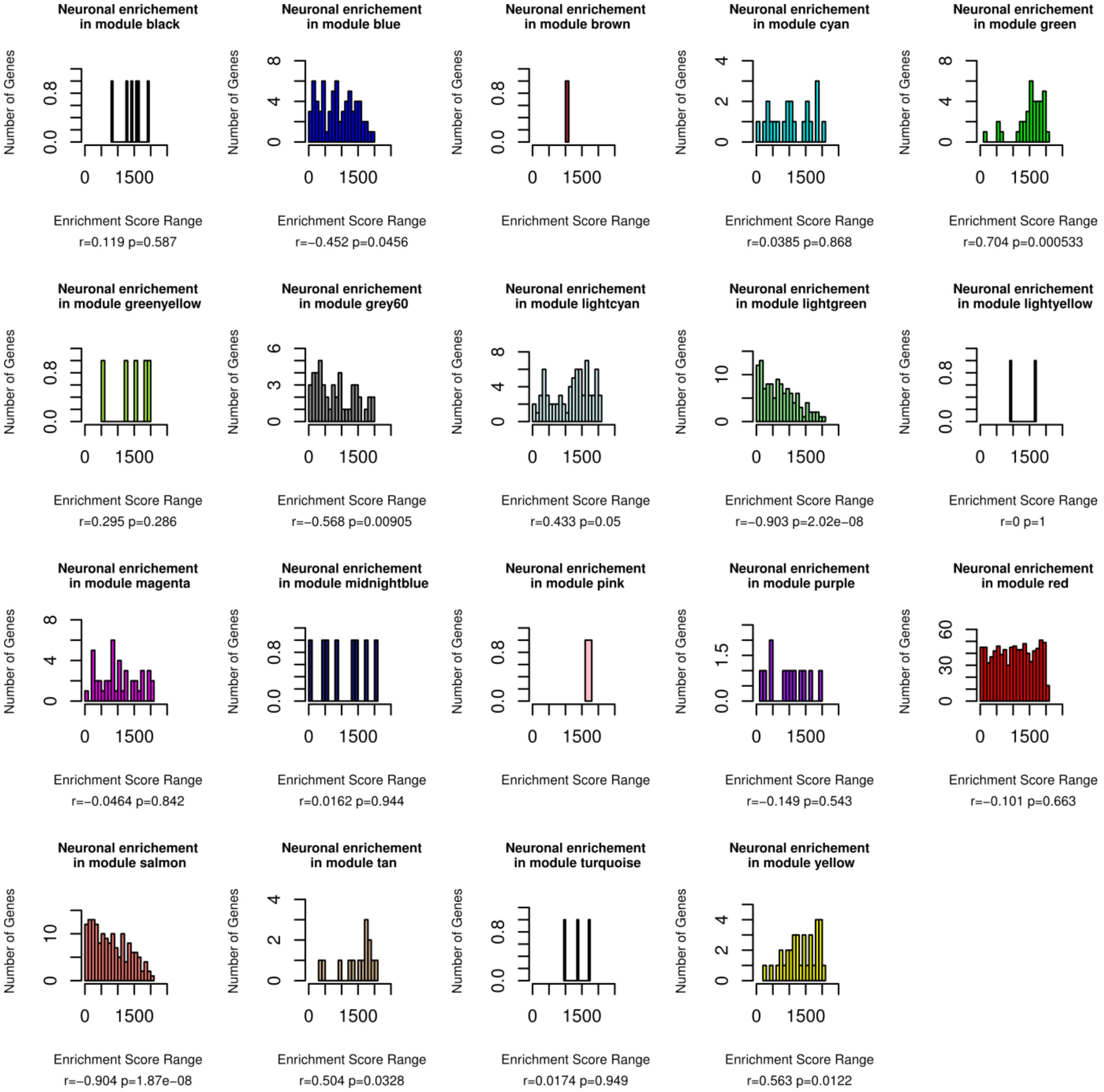

(B) Enrichment for astrocyte specific genes

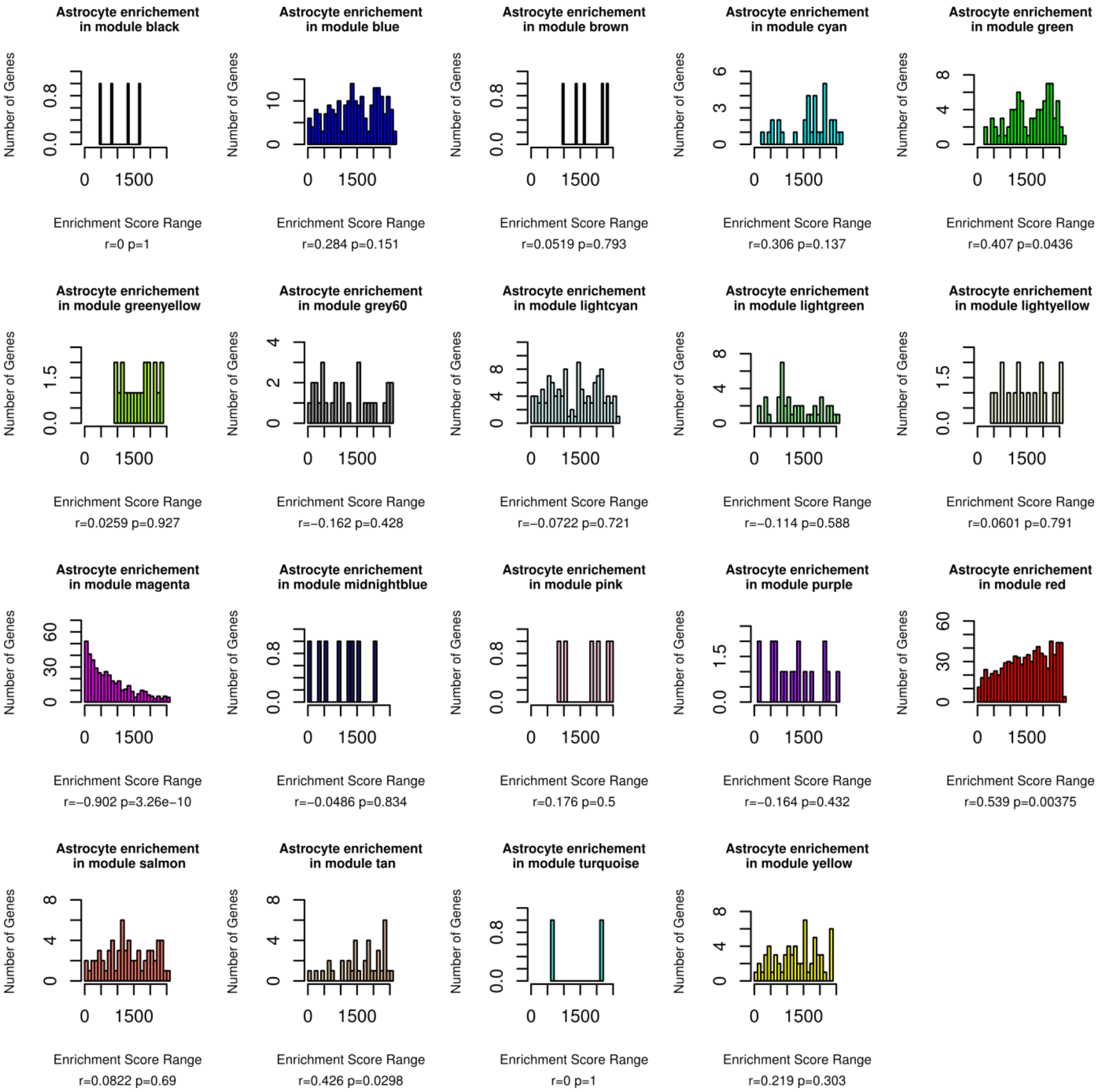

(C) Enrichment for oligodendrocytes specific genes

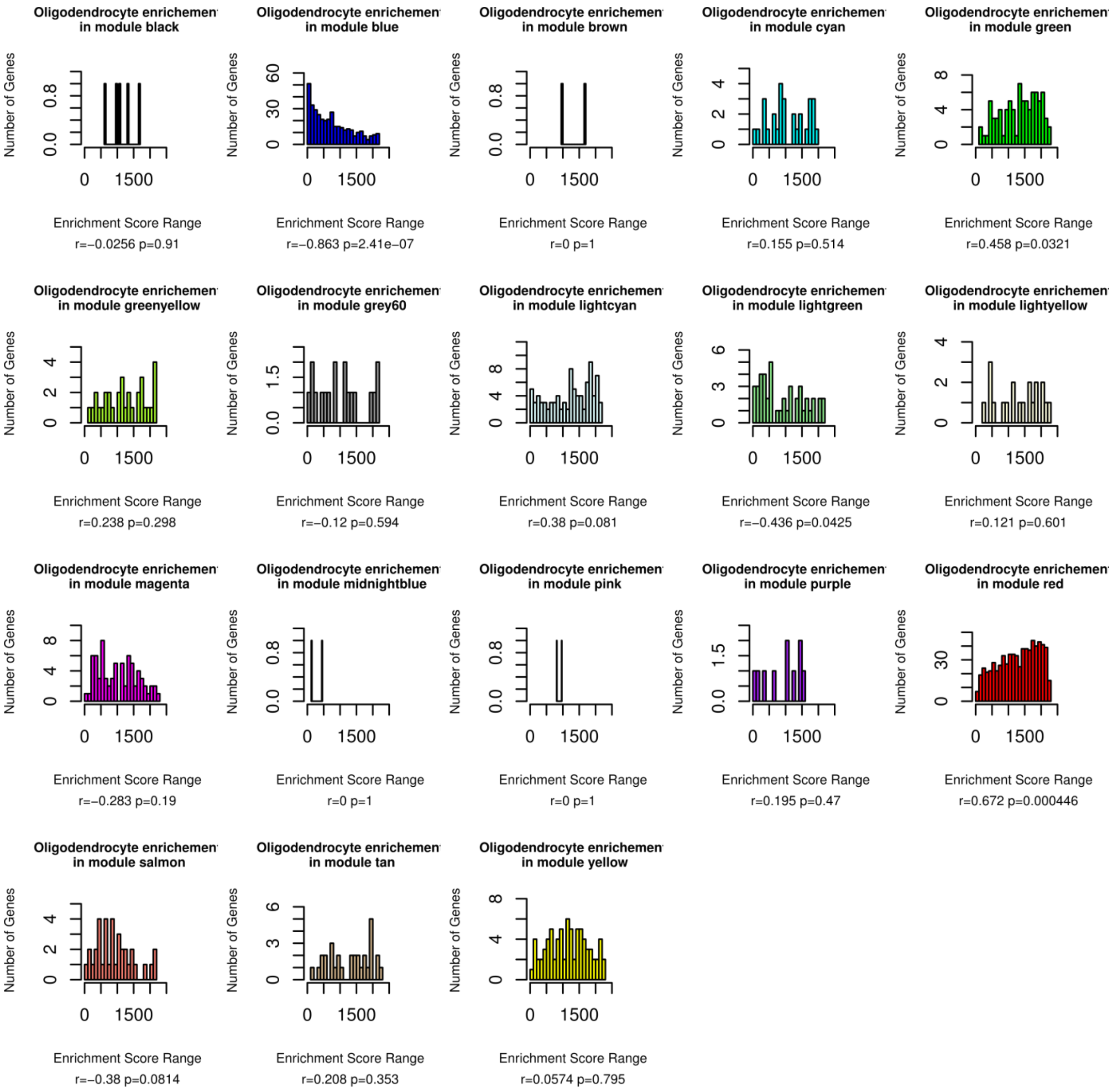

Supplement: Figure S3 — Enrichment for specific cell types in the different modules. Histogram of the rank of enrichment score for genes found to be enriched in (A) neurons, (B) astrocytes, and (C) oligodendrocytes is plotted for each module. Higher density towards the lower end of the spectrum denotes enrichment for the higher ranked cell-type genes. Pearson correlations along with P-values are listed below each plot. (A) The Salmon and Lightgreen modules show highly significant enrichment for the higher ranking of neuronal specific genes. (B) The Magenta module shows highly significant enrichment for the higher ranking astrocyte specific genes. (C) The Blue module shows highly significant enrichment for the high ranking oligodendrocyte specific genes. (PDF) [file pgen.1002556.s003.pdf]
