# Supplementary figures and images for: Networks of Neuronal Genes Affected by Common and Rare Variants in Autism Spectrum Disorders
Source: PLoS Genet. 2012 Mar 8;8(3):e1002556. doi: 10.1371/journal.pgen.1002556 (PMC3297570; doi:10.1371/journal.pgen.1002556)

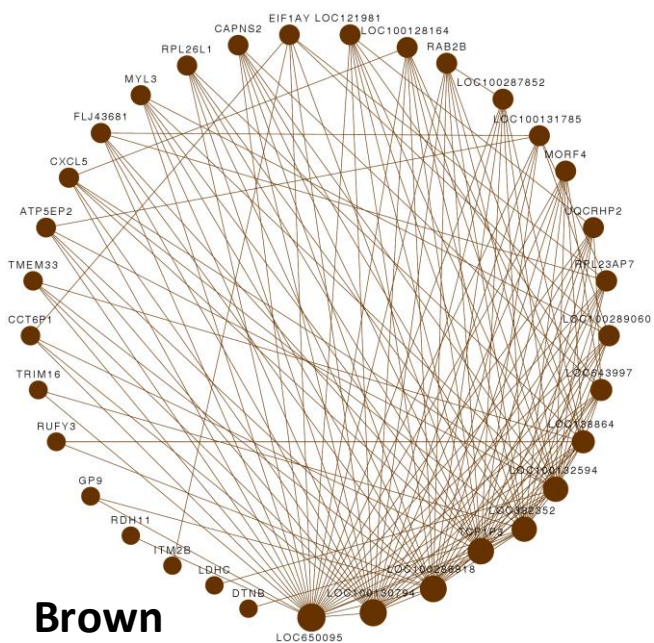

**Brown**

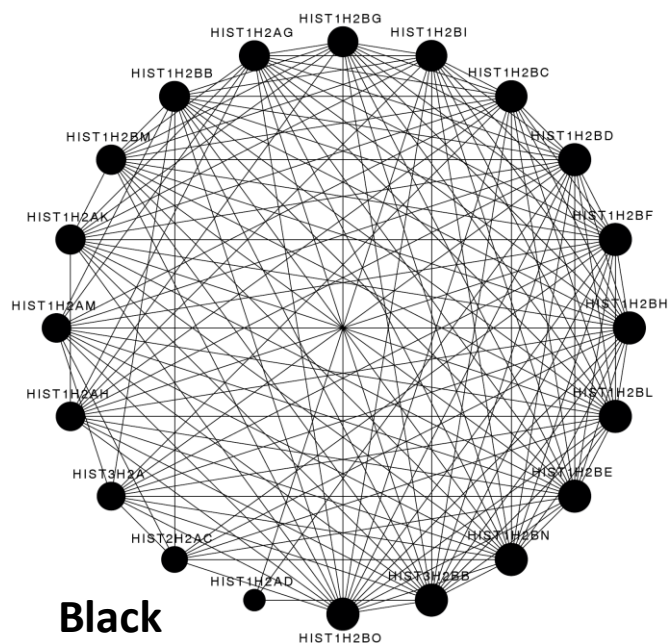

**Black**

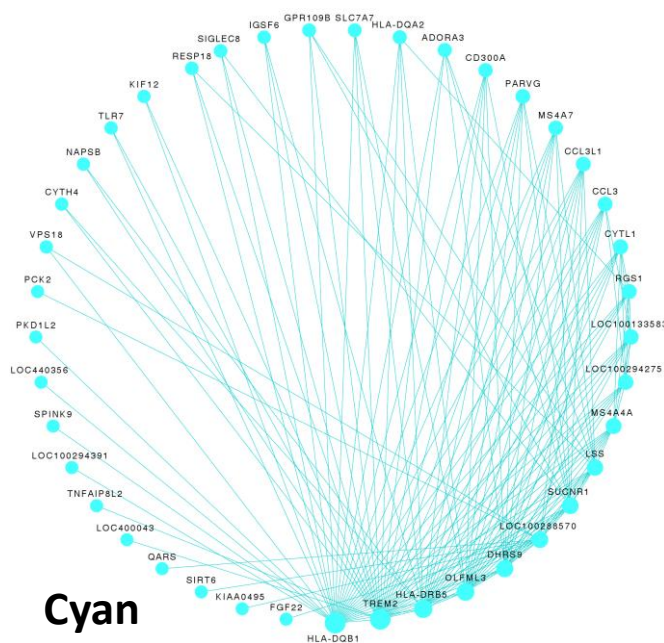

**Cyan**

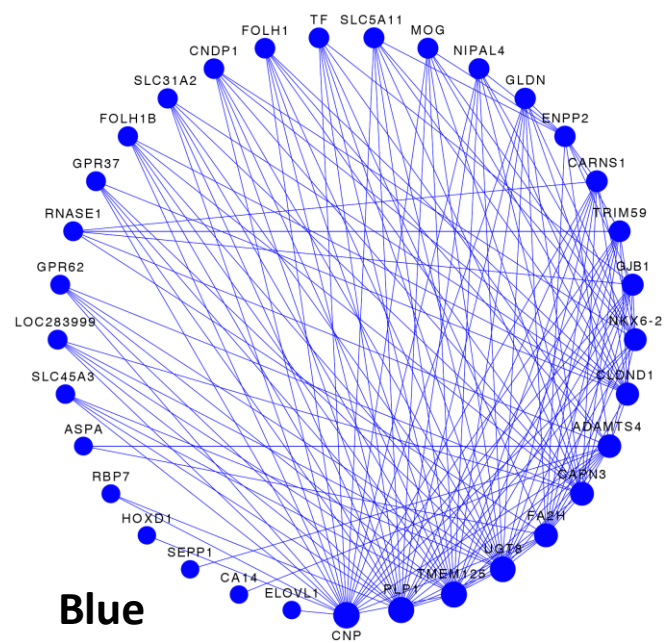

**Blue**

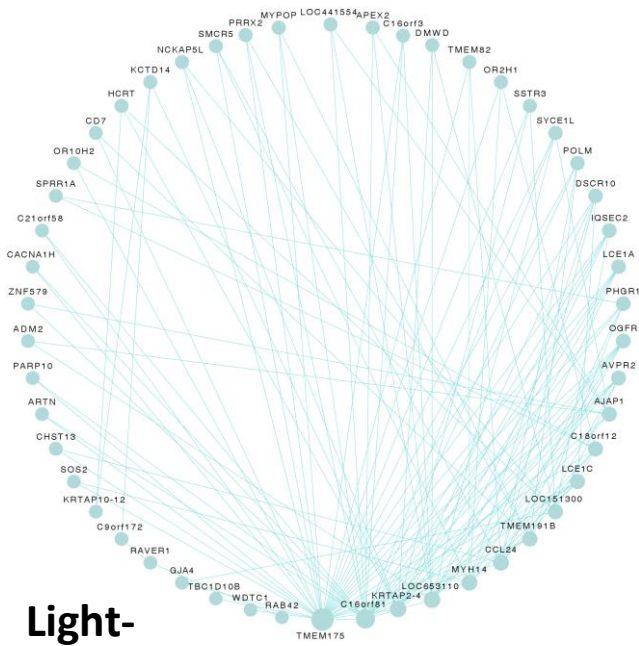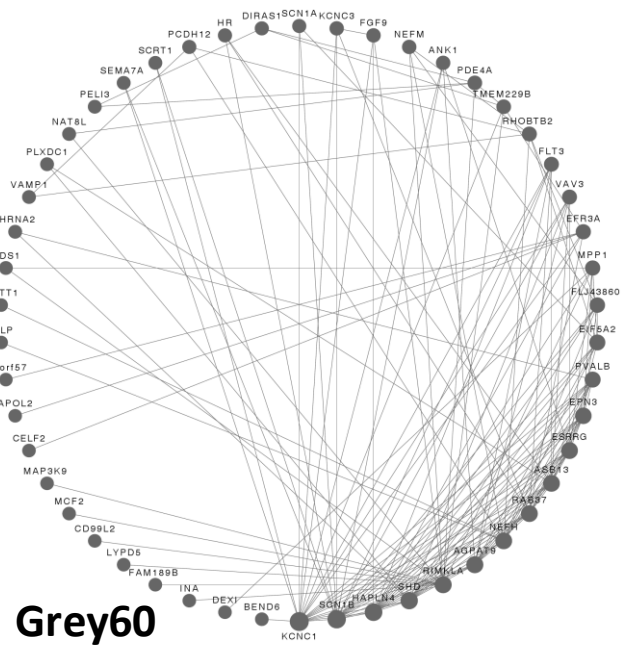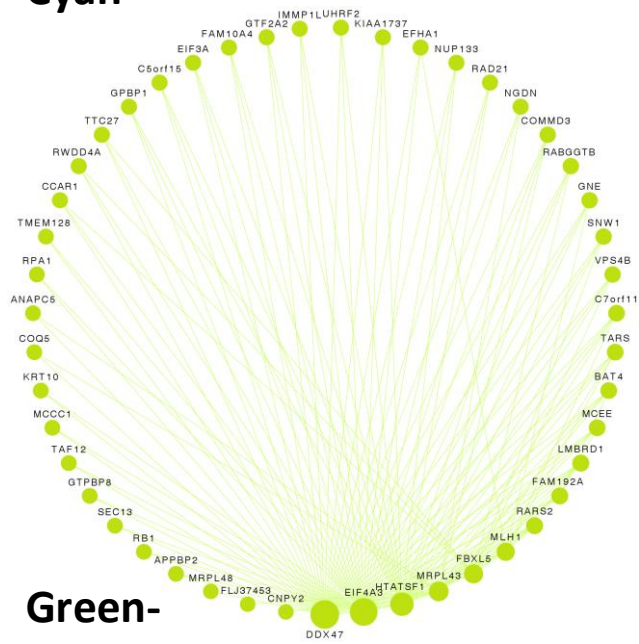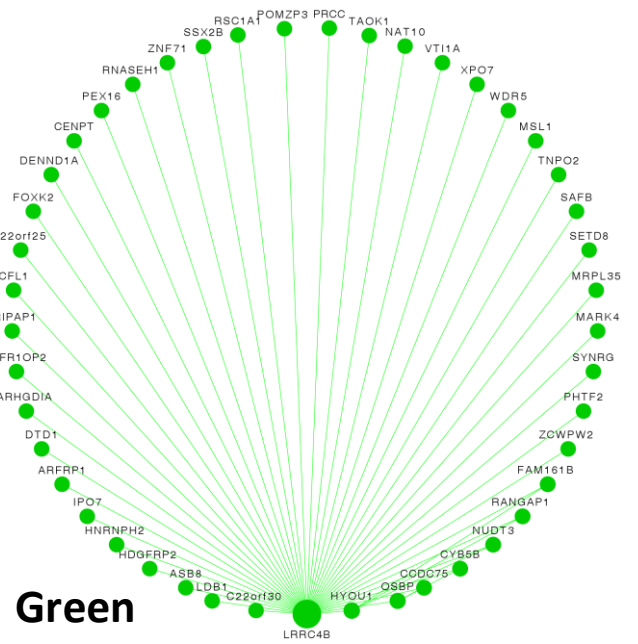

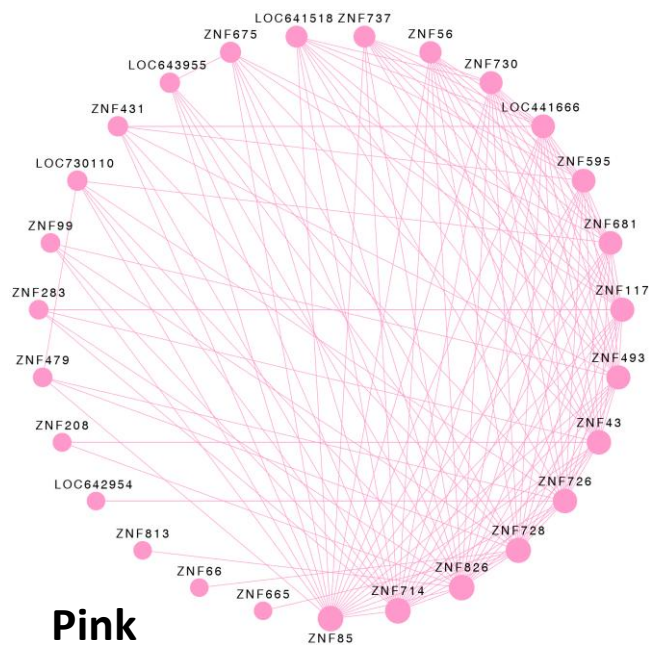

**Pink**

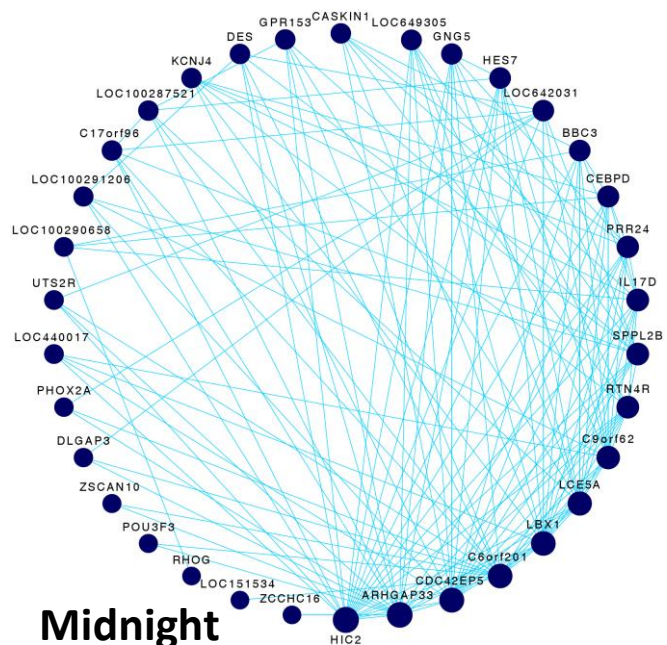

**Midnight-Blue**

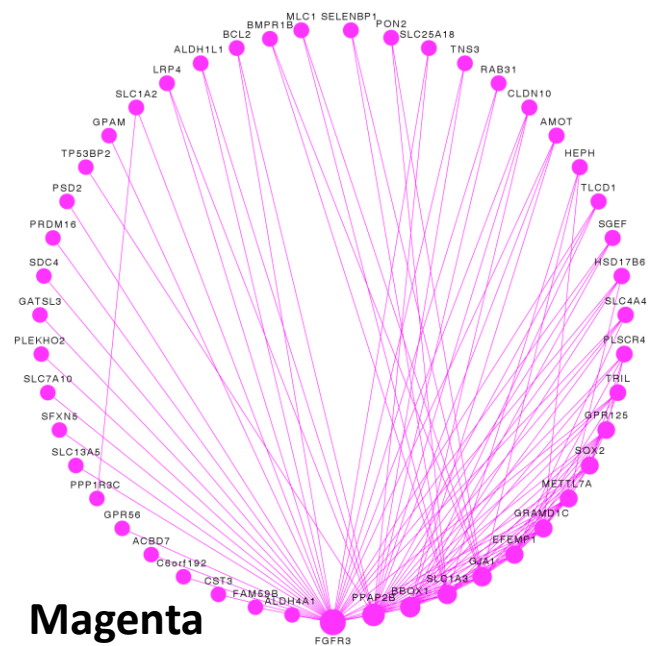

**Magenta**

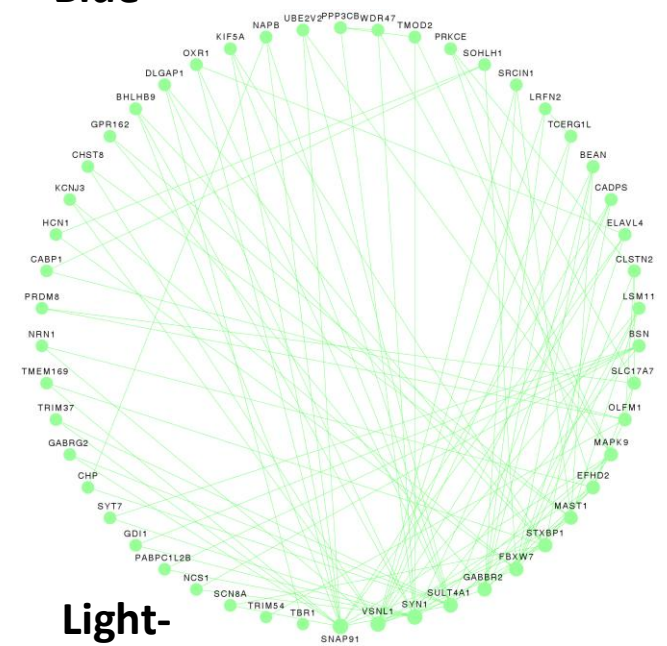

**Light-Green**

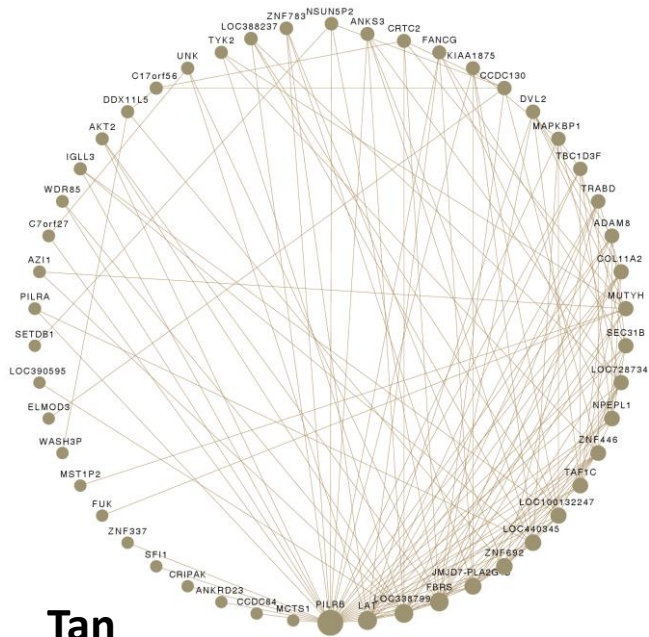

Tan

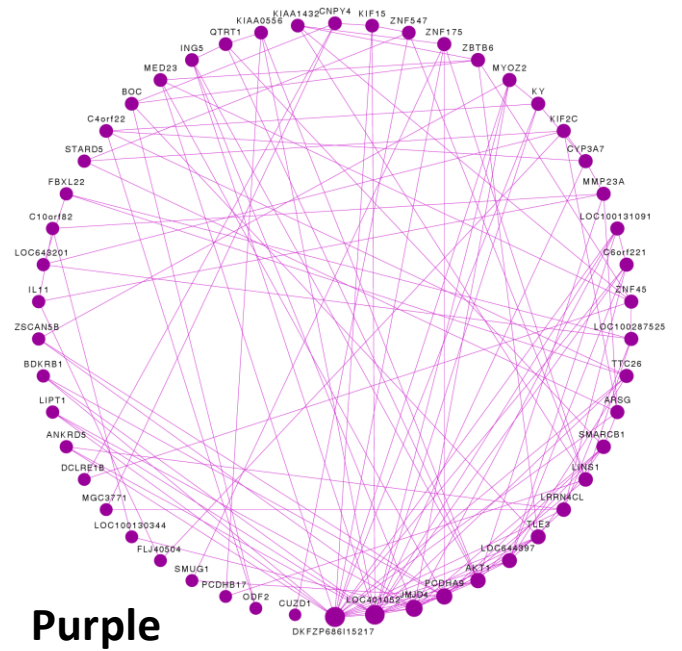

Purple

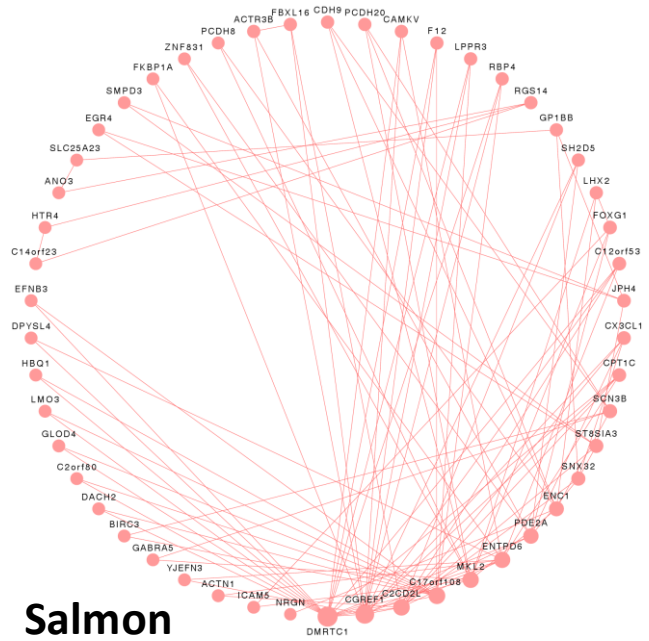

Salmon

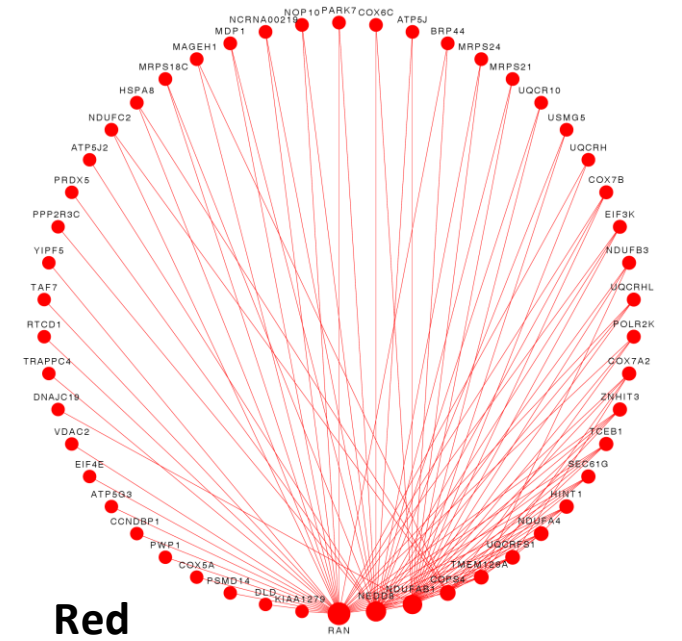

Red

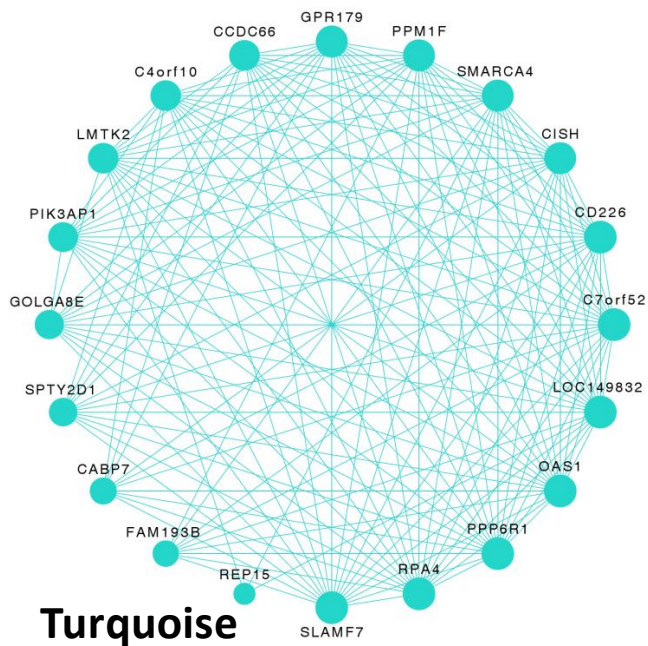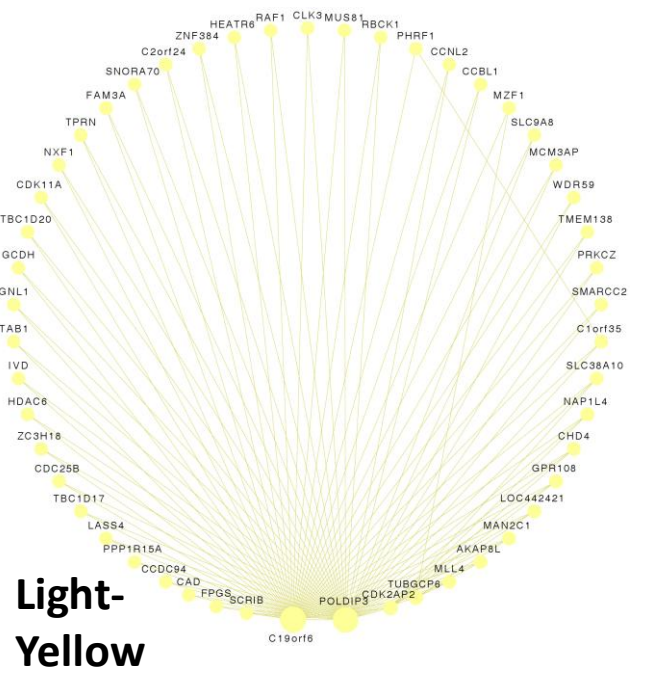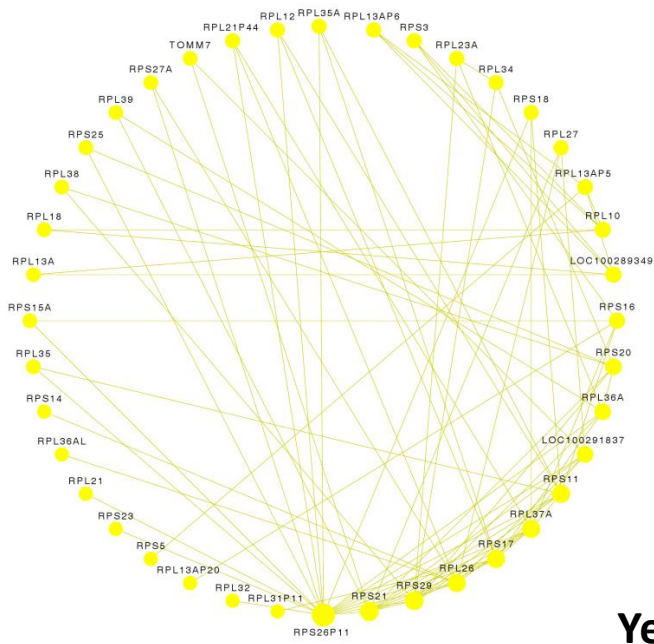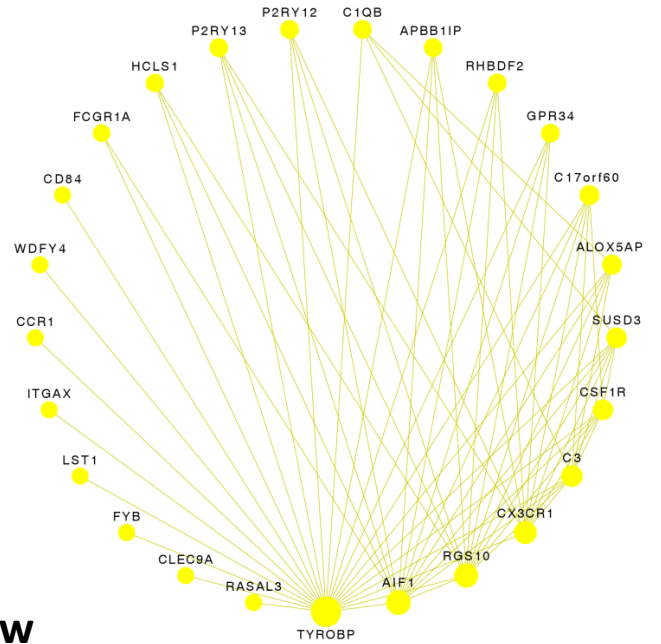

Supplement: Figure S4 — Top connections in the WGCNA modules. The top 150 connections in each module are visualized using the Cytoscape Software Package. Nodes are ordered and sized according to their degree. (PDF) [file pgen.1002556.s004.pdf]

**A**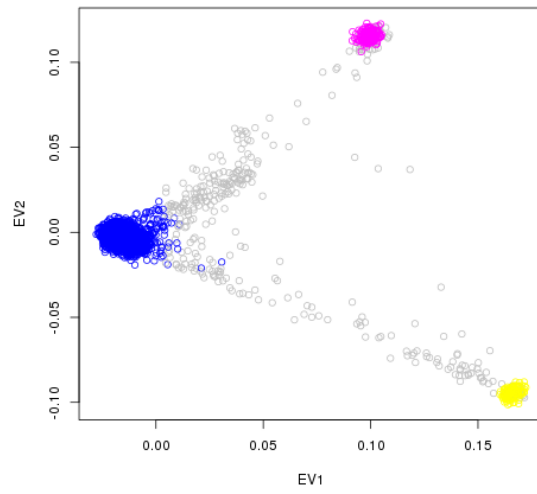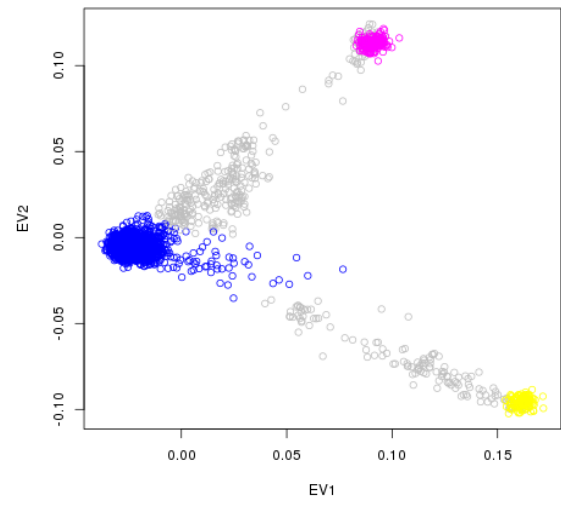**B**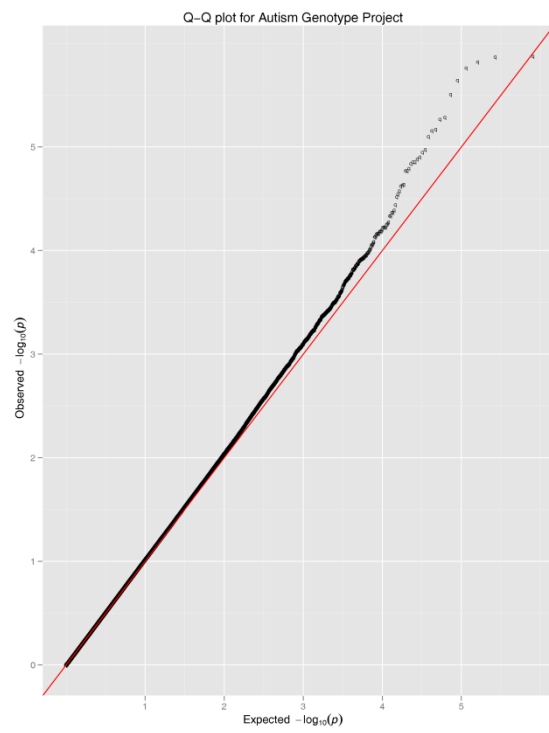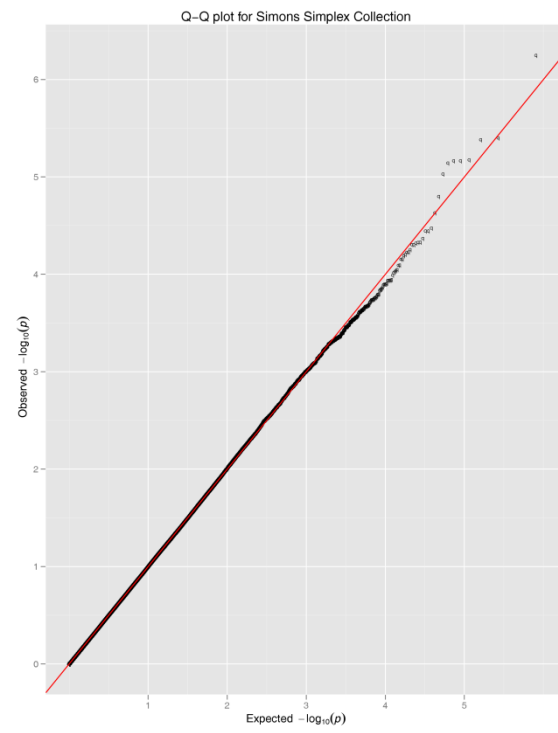

Supplement: Figure S5 — Quality control measures for GWAS. MDS clustering (A) and Q-Q (B) plots are shown for the AGP (left) and SSC (right) cohorts. (A) An MDS plot was generated incorporating samples from the HapMap Project Phase III. On this, clustering was performed using the Mclust R package. Samples (shown in gray) which did not cluster with the HapMap Caucasian (CEU) cohort (shown in turquoise) were removed from analysis. Han Chinese and Japanese samples are in purple, and Yorubans are in yellow. (B) Q-Q plot was generated by plotting the observed −log10 P against the expected under a uniform P-value distribution. (PDF) [file pgen.1002556.s005.pdf]
