# Supplementary material for: Networks of Neuronal Genes Affected by Common and Rare Variants in Autism Spectrum Disorders
Source: PLoS Genet. 2012 Mar 8;8(3):e1002556. doi: 10.1371/journal.pgen.1002556 (PMC3297570; doi:10.1371/journal.pgen.1002556)
Supplement: Figure S2 — Expression patterns of the modules eigengene across brain regions. For each module in the network, the level of the module eigengene is shown for 100 representative regions. The 100 regions were chosen using the following algorithm: first, the region showing the highest standard deviation was chosen, and then the 99 additional regions were chosen by taking recursively the region showing the lowest r2 with the previous ones. (PDF) [file pgen.1002556.s002.pdf]

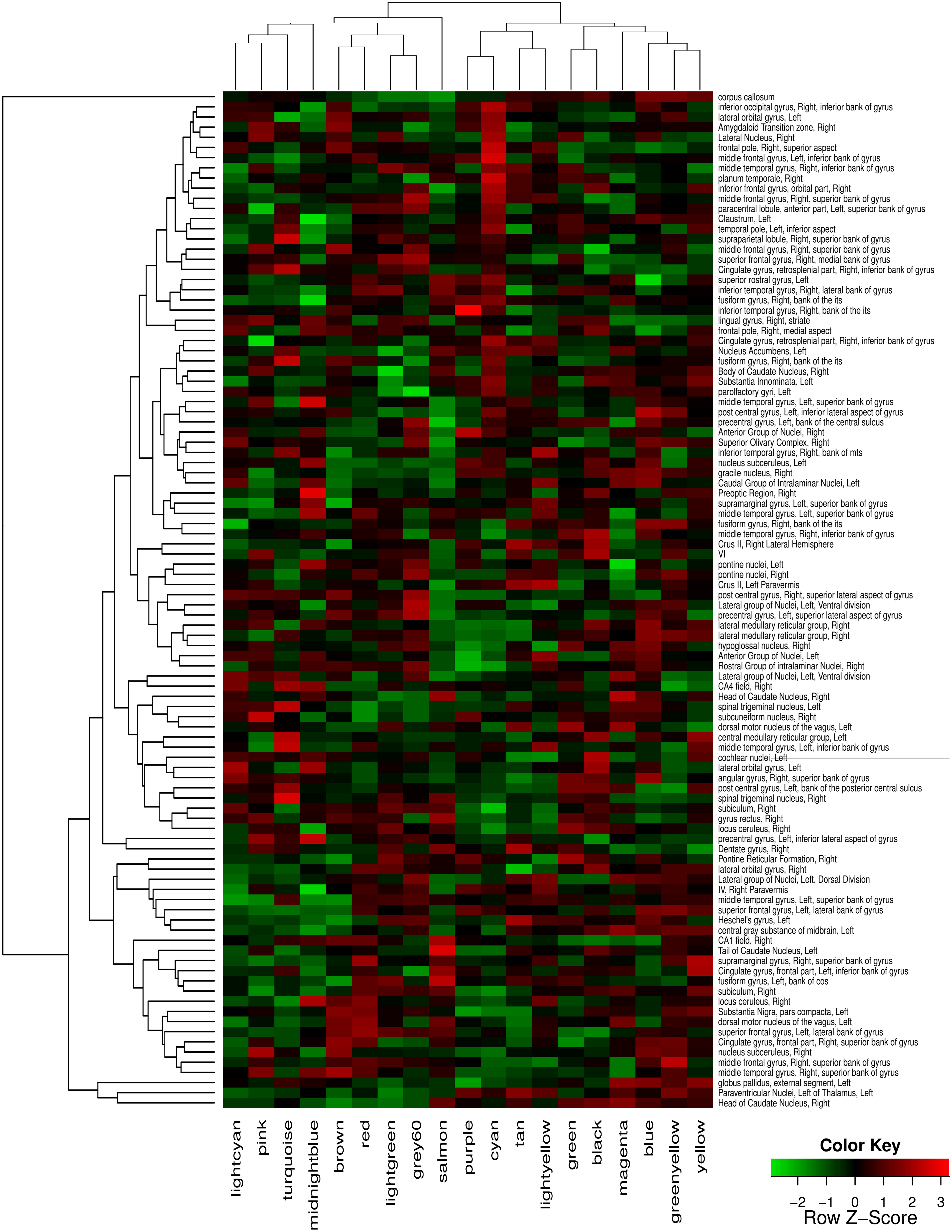

corpus callosum
inferior occipital gyrus, Right, inferior bank of gyrus
lateral orbital gyrus, Left
Amygdaloid Transition zone, Right
Lateral Nucleus, Right
frontal pole, Right, superior aspect
middle frontal gyrus, Left, inferior bank of gyrus
middle temporal gyrus, Right, inferior bank of gyrus
planum temporale, Right
inferior frontal gyrus, orbital part, Right
middle frontal gyrus, Right, superior bank of gyrus
paracentral lobule, anterior part, Left, superior bank of gyrus
Claustrum, Left
temporal pole, Left, inferior aspect
supraparietal lobule, Right, superior bank of gyrus
middle frontal gyrus, Right, superior bank of gyrus
superior frontal gyrus, Right, medial bank of gyrus
Cingulate gyrus, retrosplenial part, Right, inferior bank of gyrus
superior rostral gyrus, Left
inferior temporal gyrus, Right, lateral bank of gyrus
fusiform gyrus, Right, bank of the its
inferior temporal gyrus, Right, bank of the its
lingual gyrus, Right, striate
frontal pole, Right, medial aspect
Cingulate gyrus, retrosplenial part, Right, inferior bank of gyrus
Nucleus Accumbens, Left
fusiform gyrus, Right, bank of the its
Body of Caudate Nucleus, Right
Substantia Innominata, Left
parolfactory gyri, Left
middle temporal gyrus, Left, superior bank of gyrus
post central gyrus, Left, inferior lateral aspect of gyrus
precentral gyrus, Left, bank of the central sulcus
Anterior Group of Nuclei, Right
Superior Olivary Complex, Right
inferior temporal gyrus, Right, bank of mts
nucleus subceruleus, Left
gracile nucleus, Right
Caudal Group of Intralaminar Nuclei, Left
Preoptic Region, Right
supramarginal gyrus, Left, superior bank of gyrus
middle temporal gyrus, Left, superior bank of gyrus
fusiform gyrus, Right, bank of the its
middle temporal gyrus, Right, inferior bank of gyrus
Crus II, Right Lateral Hemisphere
VI
pontine nuclei, Left
pontine nuclei, Right
Crus II, Left Paravermis
post central gyrus, Right, superior lateral aspect of gyrus
Lateral group of Nuclei, Left, Ventral division
precentral gyrus, Left, superior lateral aspect of gyrus
lateral medullary reticular group, Right
lateral medullary reticular group, Right
hypoglossal nucleus, Right
Anterior Group of Nuclei, Left
Rostral Group of intralaminar Nuclei, Right
Lateral group of Nuclei, Left, Ventral division
CA4 field, Right
Head of Caudate Nucleus, Right
spinal trigeminal nucleus, Left
subcuneiform nucleus, Right
dorsal motor nucleus of the vagus, Left
central medullary reticular group, Left
middle temporal gyrus, Left, inferior bank of gyrus
cochlear nuclei, Left
lateral orbital gyrus, Left
angular gyrus, Right, superior bank of gyrus
post central gyrus, Left, bank of the posterior central sulcus
spinal trigeminal nucleus, Right
subiculum, Right
gyrus rectus, Right
locus ceruleus, Right
precentral gyrus, Left, inferior lateral aspect of gyrus
Dentate gyrus, Right
Pontine Reticular Formation, Right
lateral orbital gyrus, Right
Lateral group of Nuclei, Left, Dorsal Division
IV, Right Paravermis
middle temporal gyrus, Left, superior bank of gyrus
superior frontal gyrus, Left, lateral bank of gyrus
Heschel's gyrus, Left
central gray substance of midbrain, Left
CA1 field, Right
Tail of Caudate Nucleus, Left
supramarginal gyrus, Right, superior bank of gyrus
Cingulate gyrus, frontal part, Left, inferior bank of gyrus
fusiform gyrus, Left, bank of cos
subiculum, Right
locus ceruleus, Right
Substantia Nigra, pars compacta, Left
dorsal motor nucleus of the vagus, Left
superior frontal gyrus, Left, lateral bank of gyrus
Cingulate gyrus, frontal part, Right, superior bank of gyrus
nucleus subceruleus, Right
middle frontal gyrus, Right, superior bank of gyrus
middle temporal gyrus, Right, superior bank of gyrus
globus pallidus, external segment, Left
Paraventricular Nuclei, Left of Thalamus, Left
Head of Caudate Nucleus, Right
lightcyan
pink
turquoise
midnightblue
brown
red
lightgreen
grey60
salmon
purple
cyan
tan
lightyellow
green
black
magenta
blue
greenyellow
yellow
Color Key
−2
−1
0
1
2
3
Row Z−Score
